# Bumble Bees and Honey Bees on Islands Harbour Reduced Viral Species Richness, Yet Honey Bee Populations Are Dominated by a Deformed Wing Virus Recombinant

**DOI:** 10.1111/mec.70070

**Published:** 2025-08-11

**Authors:** Jana Dobelmann, Lena Wilfert

**Affiliations:** ^1^ Institute of Evolutionary Ecology and Conservation Genomics University of Ulm Ulm Germany

**Keywords:** bee, deformed wing virus, island, varroa, virome

## Abstract

Pollinators harbour diverse RNA viromes that play a vital role in their health. Yet, factors that shape viral communities are often unclear. The European honey bee ( *Apis mellifera* ) is experiencing a viral epidemic since the emergence of the parasitic mite *Varroa destructor* (varroa) introduced vector‐borne transmission, which has also been linked to increased viral spillover into wild pollinator communities. Varroa‐free island populations provide natural laboratories to study the effect of varroa, while also allowing us to ask how islands affect viral communities. Barriers that restrict the dispersal of wild pollinators and their pathogens to islands may be overcome by human‐mediated transport in managed honey bees. Here we used islands with and without varroa and matched mainland populations of honey bees ( *A. mellifera* ) and bumble bees ( *Bombus terrestris* ) from 2015 and 2021 to explore how varroa presence and island location affect the virome of managed and wild bees. We find lower viral richness on islands in both species. Bumble bees harbour a distinct viral community that was not affected by varroa but geographically structured. In honey bees, however, varroa‐present populations contained more viral reads driven by a high abundance of deformed wing virus (DWV). Within the 6 years between the sampling events, DWV underwent a shift from mostly DWV‐B towards a mix of DWV‐B and recombinant strains. Surprisingly, these shifts appeared independent of varroa. Viewing pollinator virome composition within an ecological framework provides valuable insights into the barriers to virus spread and could help to predict drivers of disease emergence.

## 1 Introduction

Insects harbour microbial communities with a diverse range of RNA viruses (Belda et al. 2019; Webster et al. 2015; Wu et al. 2020). These RNA viruses play an important role in the health of pollinators (McMenamin and Flenniken 2018), a group that has globally been in decline (Biesmeijer et al. 2006; Potts et al. 2010). Understanding viral ecology can be crucial in mitigating health threats to these insects key for maintaining food security and biodiversity. Insights into temporal and geographic patterns of prevalence can help to identify emerging diseases (Jones et al. 2008; Reperant 2010), as global mobility, trade, and travel have all contributed to disease outbreaks worldwide (Baker et al. 2022). The drivers of epidemiological patterns in pollinator populations are not well known, but islands can provide useful laboratories to study disease patterns. Pathogens seem to follow the predictions of the equilibrium theory of island biogeography (MacArthur and Wilson 1967), with fewer species in smaller, more isolated areas (Jean et al. 2016). This island effect is stronger for pathogens with a non‐human primary host than those primarily infecting humans (Jean et al. 2016), suggesting that mobile species can share high similarity in viruses over large areas (Brown et al. 2009).

The Western honey bee ( *Apis mellifera* ) is a highly mobile species, traded globally for honey production and pollination. Since the host switch of the ectoparasitic mite *Varroa destructor* (hereafter varroa) from the Eastern honey bee *A. cerana* to *A. mellifera* at the beginning of the last century (Rosenkranz et al. 2010; Traynor et al. 2020), human‐mediated transport has led to the global spread of varroa (Owen 2017). By feeding on the bees' haemolymph (Annoscia et al. 2019) and fat tissue (Ramsey et al. 2019), varroa introduced a novel transmission route for viruses (Ryabov et al. 2014). This vector‐mediated transmission is tightly linked to the emergence of the deformed wing virus (DWV) (Hasegawa et al. 2023; Wilfert et al. 2016), a formerly benign virus that has become highly prevalent (de Miranda and Genersch 2010; Nazzi et al. 2012) and is associated with increased over‐winter hive mortality (Dainat et al. 2012; Natsopoulou et al. 2017). Out of the four known DWV master variants (de Miranda et al. 2022; Mordecai, Wilfert, et al. 2016), the most common types A and B diverged ca. 300 years ago (Hasegawa et al. 2023). The formerly predominant DWV‐A, however, is currently being replaced by DWV‐B and recombinant strains (Grindrod et al. 2021; Hesketh‐Best et al. 2024; Paxton et al. 2022; Sircoulomb et al. 2025), which may be more virulent and damaging to bees (McMahon et al. 2016). The varroa‐associated declines in bee health (Bruckner et al. 2023; Traynor et al. 2016) have led to great efforts in surveying bee viruses, which revealed similar viral communities across large geographical scales (Beaurepaire et al. 2020).

With varroa having invaded most large *A. mellifera* populations, the varroa‐affected virome is relatively well studied (Brutscher et al. 2015; Chen and Siede 2007; Galbraith et al. 2018; McMenamin and Genersch 2015), although new viruses are constantly being discovered (Doublet et al. 2025; Kadlečková et al. 2024; Remnant et al. 2017; Ryabov et al. 2023). The varroa‐naïve virome, however, may look different. In Australia, for instance, where varroa was absent until 2022 (Phaboutdy and Ward 2024), honey bees harbour a diverse Picornavirales community and DWV is mostly absent (Roberts et al. 2018). Other varroa‐naïve populations have DWV, but studies commonly target few viral species (Dobelmann et al. 2024; Doublet et al. 2024, 2017; Manley et al. 2020, 2019; Martin et al. 2012; Mondet et al. 2014), so it is not known whether varroa affects virome diversity (but see Manley et al. 2020). Additionally, varroa presence could indirectly affect the virome of other pollinators. Many wild pollinators harbour viruses that are typically associated with honey bees, including DWV (Alger et al. 2019; Graystock et al. 2014; Levitt et al. 2013; McMahon et al. 2015; Radzevičiūtė et al. 2017; Singh et al. 2010), and high viral titres in varroa‐parasitized honey bees increase the likelihood of inter‐species transmission. This virus spillover from honey bees is not limited to populations with varroa (Brettell et al. 2020), but its presence has been shown to increase the prevalence of honey‐bee‐associated viruses in bumble bees (Dobelmann et al. 2024; Manley et al. 2020, 2019) and other wild insects (Loope et al. 2019; Santamaria et al. 2018). Recent studies focusing on bumble bee viromes have shown the dominance of bumble bee‐specific viruses (Doublet et al. 2025; Pascall et al. 2021; Robinson et al. 2024; Schoonvaere et al. 2018), suggesting that honey bee viruses may represent a minor component of their virome. Few honey bee viruses are actively replicating in bumble bee hosts in field studies (Doublet et al. 2025) and DWV strain diversity suggests repeated spillover but no onward transmission from bumble bees (McKeown et al. 2025). In laboratory experiments, transmission within bumble bees appears to be inefficient (Streicher et al. 2024; Tehel et al. 2022). Yet, DWV can replicate in bumble bees under laboratory conditions (Gusachenko et al. 2020), but also in the field (Manley et al. 2019) and may interact with bumble bee viruses. How vector‐borne transmission through varroa has shaped the honey bee virome, and with it the virome of wild species that forage in the same environment remains unknown.

In this study, we use multiple varroa‐free and varroa‐present island locations and the varroa‐present mainland in the British Isles and in France to study the effects of varroa presence, island location, and time on the RNA virome in bees. We contrast *A. mellifera*, a managed species that is directly affected by varroa‐mediated virus transmission, with wild *Bombus terrestris* , which share foraging resources but do not have vector‐mediated virus transmission. Wild bumble bee populations are expected to have a less diverse virome on islands, but in managed honey bees, human‐mediated transport may erode barriers for pathogen spread. We hypothesise that varroa affects the species composition and temporal stability of honey bee viromes and that virus spill‐over from varroa‐infested honey bee populations affects the viral community in bumble bees. Using these rare varroa‐free islands can provide a glimpse into the ancestral pre‐varroa community and varroa‐independent DWV variant shifts, additionally allowing us to gain insights into the geographical distribution of wild bee viruses and the impact of varroa on bee‐virus ecology.

## 2 Material and Methods

### 2.1 Sampling and RNA Extraction

**FIGURE 1:**
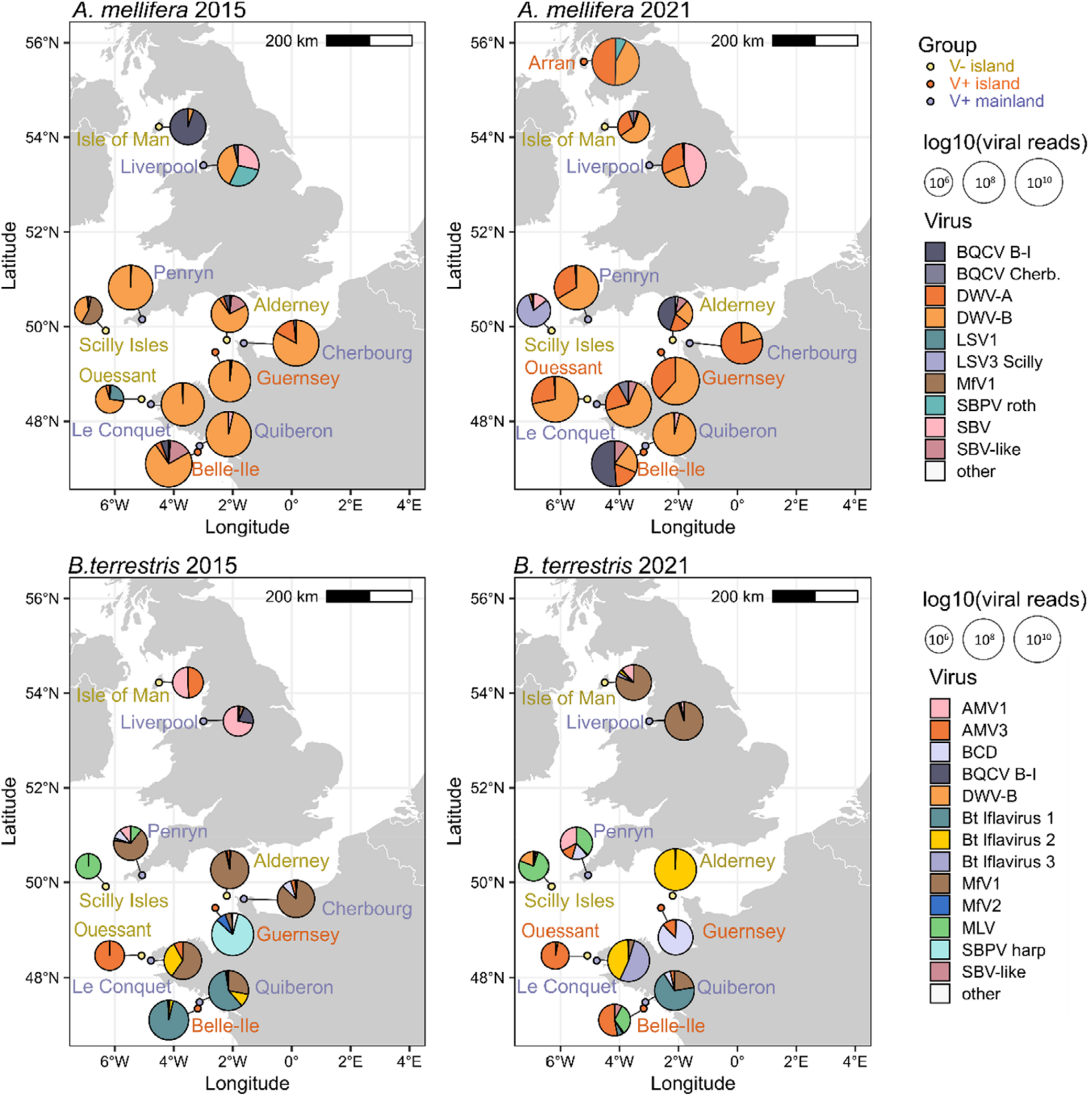
Maps showing *Apis mellifera* (top) and *Bombus terrestris* (bottom) virome composition in 2015 (left) and 2021 (right). The coloured label of the sampling sites indicates whether bees were from a varroa‐free (V−) island (dark yellow), a varroa‐present (V+) island (orange), or the V+ mainland (purple). *Note that Ouessant was V− in 2015 but V+ in 2021. Pie charts show the relative abundance of the most common viruses in both host species (colours), and the size indicates the normalised number of viral reads (log10 transformed) in each sample. Viruses in *A. mellifera* include: Black queen cell virus (BQCV) Belle‐Ile and Cherbourg variant, deformed wing virus type A and B (DWV‐A and DWV‐B), Lake Sinai virus 1 and 3 Scilly variant (LSV1 and LSV3 Scilly), Mayfield virus 1 (MfV1), slow bee paralysis virus strain Harpenden and Rothamsted (SBPV roth and harp), sacbrood virus (SBV) and SBV‐like virus. Viruses in *B. terrestris* were: Allemuir hill virus 1 and 3 (AMV1 and AMV3), Bombus cryptarum densovirus (BCD), BQCV Belle‐Ile variant, DWV‐B, three novel Iflaviridae (Bt Iflavirus 1 to 3), Mayfield virus 1 and 2 (MfV1 and MfV2), Mill lade virus (MLV), SBPV harp, SBV‐like virus.

*A. mellifera* and *B. terrestris* foragers were collected from flowers in the summer of 2015 as described by Manley et al. (2019) and in the summer of 2021 at 12 sites on the British Isles and in France (Figure 1 and Table S1). Sites were grouped by varroa presence, that is, varroa‐free (V−) or varroa‐present (V+), and into island or mainland sites. V− islands were Alderney, the Isle of Man, Ouessant, and the Scilly Isles; V+ islands were Arran, Belle‐Ile, and Guernsey; and the V+ mainland included Cherbourg, Le Conquet, Liverpool, Penryn, and Quiberon (Figure 1). The French island of Ouessant was V− in 2015, but varroa was introduced to the island before the second sampling in 2021 (Dobelmann et al. 2024; Ménage and L'Hostis 2021). In Cherbourg, only *A. mellifera* could be collected in 2021, and on Arran, only *A. mellifera* was obtained and only in 2021, resulting in 44 sampling pools. Samples were frozen within 24 h of collection and transported to the lab on dry ice or in a dry shipper.

### 2.2 Pools for RNA‐Seq

Individual bees were laterally dissected and homogenised in a FisherBrand Bead Mill 24 (speed 5 m/s for 25 s with 3 cycles and 20 s pause in‐between). RNA was extracted from one half using 1.3 mL TRI Reagent (Sigma‐Aldrich) and 1‐Bromo‐3‐chloropropane (Sigma‐Aldrich) and eluted in 80 or 120 μL H_2_O, for *A. mellifera* and *B. terrestris*, respectively. In *B. terrestris* , additionally, DNA was extracted from one hind leg using Chelex (5% wt/vol) and 20 μL proteinase K (100 μM) to distinguish the species from *B. lucorum* using a PCR with species‐specific length polymorphism (details in supplemental material S1).

RNA concentration was measured by fluorescent dye detection using the Quantiflour RNA kit (Promega). Equimolar amounts of RNA from 30 individuals for each site, year, and species were pooled for sequencing (for 6 libraries, fewer individuals were available: 13 *B. terrestris* Ouessant 2015, 17 *B. terrestris* Guernsey 2021, 22 *A. mellifera* Isle of Man 2015, 28 *A. mellifera* Alderney 2021 and Belle‐Ile 2015 and 29 *A. mellifera* Liverpool 2015). RNA integrity of sample pools was tested using the RNA Screentape on a Tapestation (Agilent) (RIN value > 9, except RIN = 8 in *A. mellifera* from Le Conquet 2015 and RIN = 7.8 in *A. mellifera* from Guernsey 2021) and RNA quality was verified using a Lunatic spectrophotometer (Unchained Labs) (260/280 > 1.8). Library preparation, including poly(A) selection and 150 bp stranded paired‐end sequencing using DNBSEQ‐T7, was performed by BGI Genomics (Shenzhen, China).

### 2.3 Bioinformatics Pipeline

Sequencing yielded 44 RNA‐seq libraries with 208–237 M reads (mean 230 M, Table S2). Reads were trimmed with sickle v1.33 (Joshi and Fass 2011), removing bases with quality below 25, and read quality was checked with fastqc v0.11.8 (Babraham Bioinformatics). To remove reads from host genomes, we aligned reads to *A. mellifera* (Amel_HAv3.1, GCA_003254395.2) and *B. terrestris* (iyBomTerr1.2, GCA_910591885.2) reference genomes in Hisat2 (Kim, Paggi, Park, Bennett, & Salzberg, 2019). Unmapped reads that contained microbial reads including viral reads were *de novo* assembled in Trinity v2.11.0 with a minimum contig length of 300 bp and a minimum kmer coverage of 2 (Grabherr et al., 2011). Contigs were used as bait in blastx searches in DIAMOND v0.8.37 (Buchfink et al. 2015) to find viral candidates, retaining a single hit for each contig. The reference database comprised of 31,706 protein reference sequences downloaded from NCBI Virus (on 15.03.22) with filtering for arthropod hosts and 500–20,000 amino acid (aa) lengths. From these, 298 contigs with at least 80% similarity to a reference and query coverage of at least 300 aa were imported into Geneious Prime v2024.0 (Kearse et al. 2012), manually grouped by taxonomy according to their best hit and then used to retrieve virus reference genomes from GenBank. These reference genomes were used to create a nucleotide database used in a second step to map reads for abundance estimation (Table S3). The two dsDNA viruses detected, Apis mellifera filamentous virus and Yalta virus, were excluded as their genomes vastly differ from the much shorter RNA and ssDNA viruses.

To improve our nucleotide reference database, we included 14 consensus sequences from *de novo* assembled contigs that were used when blastx searches indicated the presence of multiple strains of a virus or for putative novel viruses found in contigs that showed low similarity to a reference (< 80%) but high alignment coverage (> 1000 aa) (Table S4 and Table S5, GenBank accession no PV239920 to PV239933). We considered contigs as putative novel viruses when they shared < 90% aa sequence identity in the conserved RNA‐dependent RNA polymerase (RdRp) with previously described species (Table S4) (Edgar et al. 2022). For viruses with multiple strains, pairwise comparisons were used to group similar contigs (> 97% identity) before extracting consensus sequences. No two viruses in our reference database shared more than 90% nucleotide (nt) identity in the RdRp (Tables S6–S10).

The presence and abundance of viruses in each sample was determined by mapping reads that did not map to the host genome to a nucleotide reference database with 46 references containing 26 high‐confidence viral hits of known viruses, 14 *de novo* assembled virus contigs, and 6 known bee viruses (although not detected in blastx searches, Table S3). We used CoverM v0.6.1 (available at https://github.com/wwood/CoverM) with *coverm contig*, –sharded to choose the best hit for each read pair and bwa‐mem2 v2.0 (Li 2013) for mapping with a minimum 97% identity. To quantify reads and create coverage maps, we used samtools v1.10 (Li et al. 2009) with *sort* to create position‐sorted files and *depth* to count read depth at every position. Reads from viruses covering < 33% of the genome or < 75% of the RdRp (non‐structural protein 1 for ssDNA viruses) were excluded from the analyses to reduce the chance of false positives.

### 2.4 Virus Abundance and Virome Diversity

Analyses were performed in R v4.4.1 (R Core Team 2014). We normalised the abundance of reads by multiplying the number of reads by the total reads in each library, divided by the average number of reads across all libraries. We used pairwise Wilcoxon tests to compare the ratios of viral reads between *A. mellifera* and *B. terrestris* libraries, and between groups (V− islands, V+ islands, and V+ mainland) within both host species. Virus diversity was calculated as the number of viruses (richness) and the inverse Simpson's diversity index (D) using the *vegan* package (Oksanen et al. 2017). Libraries with only one viral species were excluded from alpha and beta diversity measures. Alpha diversity between host species and between groups within host species was compared using pairwise t‐tests. To assess how virus communities differ between island and mainland sites, with varroa presence, sampling year, and geographical location (latitude), we used Bray–Curtis dissimilarity to calculate viral community dissimilarity matrices for each species and ran permutational analyses of variance (PERMANOVA) with 999 permutations. Non‐metric Multidimensional Scaling (NMDS) plots were created using 4 dimensions.

### 2.5 DWV Recombinant Breakpoints

To verify the identification of potential recombinant variants between DWV‐A and DWV‐B, we used PCR and Sanger sequencing. While assembly tools can falsely create artefacts that resemble recombinant strains, targeted PCR can distinguish recombinant strains from artefacts. We designed primers spanning five potential breakpoints between DWV‐A and DWV‐B to amplify and distinguish recombinant fragments from assembly artefacts that can be created when viral strains share a high similarity. Primers were either a forward primer matching DWV‐A and a reverse primer matching DWV‐B or *vice versa*, and were designed according to *de novo* assembled contigs (Table S11). Reverse transcription of RNA pools from 2021 was carried out using random hexamers and GoScript reverse transcriptase kit (Promega) according to the manufacturer's instructions (5 min at 25°C, 1 h at 42°C, 15 min at 70°C). PCR was performed using GoTaq Flexi (Promega) with 2.5 mM MgCl_2_, 0.2 mM dNTPs, 0.5 μM each primer, 0.375 U polymerase, and 2 μL 1:10 diluted cDNA in 15 μL reactions with three technical replicates. A total of 5 μL PCR product was cleaned using 2 μL ExoSAP‐IT (Applied Biosystems) or 10 U Exonuclease I (New England Biolabs) and 5 U Antarctic Phosphatase (New England Biolabs) with incubation at 37°C for 30 min and 80°C for 15 min. Sanger sequencing was performed at Eurofins Genomics (Ebersberg, Germany). Sequences were manually trimmed, and technical replicates were checked for PCR‐mediated chimera in Geneious v2023.2.1 (Kearse et al. 2012) and then mapped to the DWV‐A and DWV‐B references (PV239925 and PV239926). Breakpoints were visually identified as regions in which sequences switched from showing high similarity to one variant to the other. As DWV‐A and DWV‐B share 85% nt identity across the genome, breakpoints can only be called as regions. To gain information on the infections of individual bees in the pools, bees from 2021 were screened for DWV‐A and DWV‐B by RT‐PCR (details in S3), and data from 2015 was retrieved from Manley et al. (2019).

## 3 Results

### 3.1 Virus Detection and Discovery

We examined the virome composition in 12 *A. mellifera* populations and 11 *B. terrestris* populations from 2015 and 2021. Populations were varroa‐free island refugia (V− island: 4 in 2015 and 3 in 2021), varroa‐present islands (V+ island: 2 in 2015 and 4 in 2021) and varroa‐present mainland sites (V+ mainland: 5 in both years) in the British Isles and France (Figure 1). In total, we found 28 viruses (20 in *A. mellifera* and 18 in *B. terrestris* ) with more than 33% of the genome and at least 75% of the RdRp (non‐structural protein 1 for ssDNA viruses) covered in at least one sample (Table S3). These viruses mostly belonged to viral families with +ssRNA genomes (Dicistroviridae, Iflaviridae, Sinhaliviridae, and unclassified Picornavirales), but also included one Rhabdoviridae with an ‐ssRNA genome, one Parvoviridae with an ssDNA genome, and multiple unclassified Riboviria. In each library, we found one to nine viruses (Figure S1 and Figure S2). Both libraries with only one viral species were from *B. terrestris* on V− islands in 2015, although another V− island in 2015 (Alderney) had seven species (Figure S2).

For some viruses, blastx searches indicated the presence of multiple strains, and we used the assembled contigs as references for read mapping. Those included two strains of black queen cell virus (BQCV Belle‐Ile [PV239920] and Cherbourg [PV239921]) which shared 88% nt identity across the genome, 89% across the RdRp (Table S6) and 95% aa identity in open reading frame (ORF) 1 and 97% in ORF2. We also detected two of the four master variants of DWV, type A [PV239925] and B [PV239926], and although blastx hits indicated high similarity (> 97% aa identity) with the protein reference database, we generated consensus sequences for both variants to improve read mapping. Those DWV‐A and DWV‐B reference sequences had 85% identity at the nucleotide level (85% RdRp identity, Table S7) and 95% at the protein level. We further found that multiple *A. mellifera* libraries harboured two contigs matching sacbrood virus (SBV), one with 95%–100% aa identity and a second with 84%–85% aa identity. Contigs matching the former were used to generate an SBV reference sequence [PV239932] that showed high similarity with SBV from Sweden [MT636329, 98% nt identity], and contigs matching the latter generated a putative novel SBV‐like virus [PV239933] that shared 76% nt identity with SBV from South Korea [OR496425] and 88% aa identity with the RdRp of an SBV isolate from Papua New Guinea [QKW94194, Table S4]. Similarity between the two references was 75% nt identity across the whole genome and 77% in the RdRp (Table S7). In the diverse group of Lake Sinai viruses (LSVs, family: *Sinhaliviridae*), we included 11 genomes in our reference database, all < 90% RdRp nt identity (Table S9). Four of these were generated from *de novo* assembled contigs that all shared < 87% aa identity to LSVs in the protein reference database. We putatively named two strains, LSV France [PV239928] and LSV Liverpool [PV239929], after their locations without a number as they were < 80% identical to LSV3 and LSV8, respectively (Table S4). The third and fourth strains, LSV TO Belle‐Ile [PV239930], which was most similar to LSV TO [NC_035116, 81% nt identity] and LSV3 Scilly [PV239927], most similar to LSV3 [MZ821904, 87% nt identity], were 80%–90% identical to known strains (Table S5) and locations were added to strain names.

The blastx search indicated the presence of Mayfield virus 2 (MfV2) in two *B. terrestris* libraries, although with 85% or 97% identity with the reference sequences [QAY29258] so that we used the consensus of the two contigs as a MfV2 reference [PV239931] for abundance estimation (Table S5). This MfV2 strain showed 94% nt identity to MfV2 [MH614305] and 82% nt identity (85% RdRp) to the other MfV in our reference database, MfV1 (Table S10). We further identified three putative novel Iflaviridae that were associated with *B. terrestris* from contigs with 50%–80% aa identity and > 1000 aa query coverage. Those contigs either matched Darwin bee virus 2 or 4 [AWK77843 or AWK77851] with 51% to 58% aa identity or SBV [ASS83209] with 79% aa identity. Pairwise comparisons indicated the presence of three viruses, and contigs with > 97% nt identity were collapsed into one consensus sequence. All three sequences were predicted to contain one ORF including the RdRp and were putatively named Bombus terrestris Iflavirus 1–3 (hereafter Bt Iflavirus 1–3, [PV239922–PV239924]). Bt Iflavirus 1 and 2 shared low similarity to an unclassified Iflaviridae sp. from China [XCO48714, 47% aa identity] and Bt Iflavirus 3 was most similar to Darwin bee virus 4 [AWK77851, 59% aa identity, Table S4]. Bt Iflavirus 1 and 2 shared 81% nt identity (82% RdRp nt identity) and 92% aa identity. The third, Bt Iflavirus 3 had only 62% nt identity (67% RdRp) with the other strains (57% at the aa level, Table S7).

### 3.2 Virus Abundance

**FIGURE 2:**
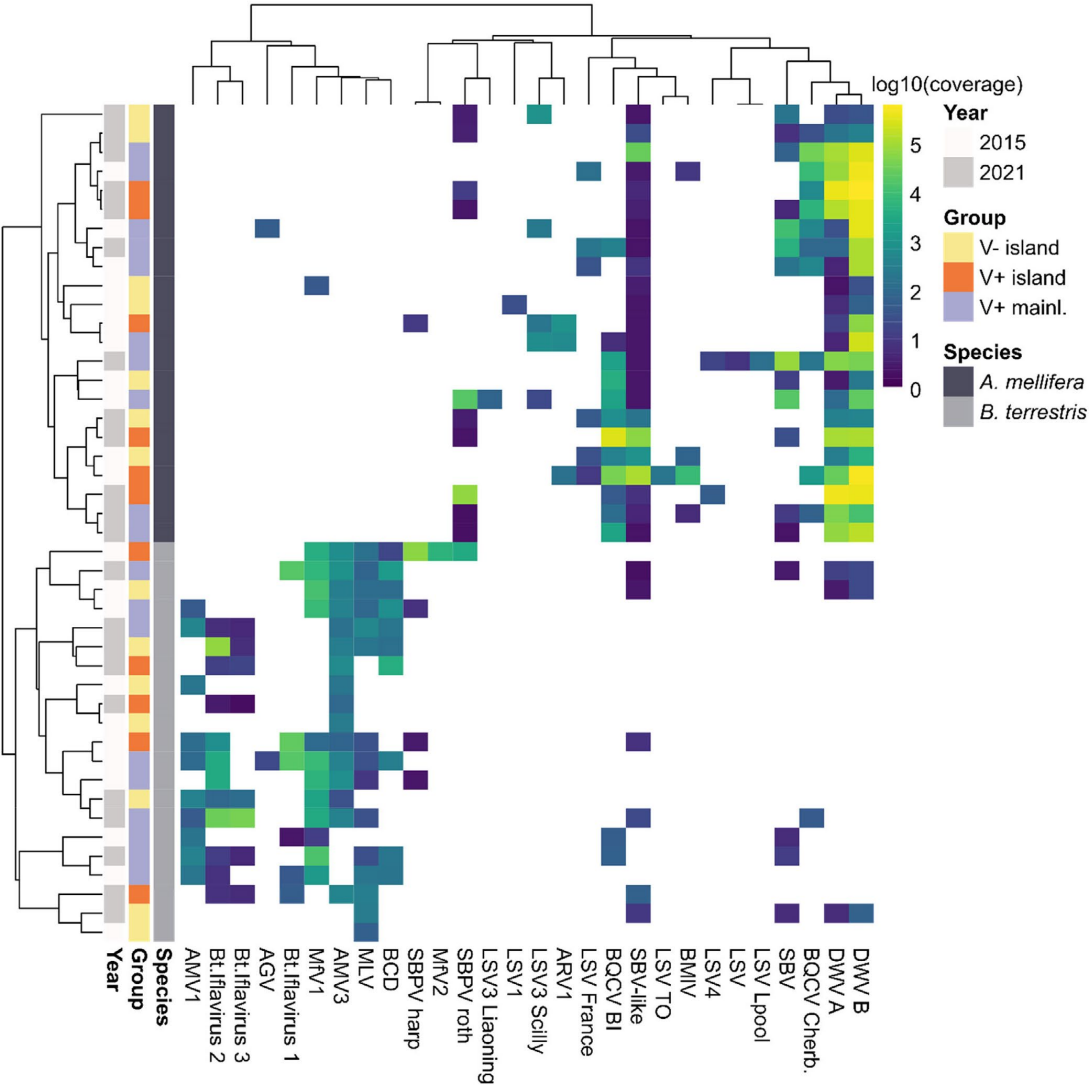
Heatmap showing hierarchical clustering of *Apis mellifera* and *Bombus terrestris* viromes represented by normalised read coverage. The colour scale shows log10 transformed coverage with yellow showing high and blue low abundance. Note that virus absence is shown in white. The colours on the left show the sampling year (white: 2015 and light grey: 2021), whether the sample was collected on a varroa‐free island (yellow: V− island), a varroa‐present island (orange: V+ island) or the V+ mainland (puple: V+ mainl.) and the host species (dark grey: *A. mellifera* and grey: *B. terrestris* ). Virus acronyms: Allermuir Hill virus 1 and 3 (AMV1, AMV3), Aphis gossypii virus (AGV), Apis rhabdovirus 1 (ARV1), Bee Macula‐like virus (BMlV), black queen cell virus strain Belle‐Ile and Cherbourg (BQCV BI and Cherb.), Bombus cryptarum densovirus (BCD), deformed wing virus A and B (DWV‐A, DWV‐B), Dumyat virus (DV), Bombus terrestris Iflavirus 1, 2 and 3 (Bt Iflavirus 1–3), Lake Sinai virus (LSV) strains (LSV, LSV1, LSV3 Liaoning, LSV3 Scilly, LSV4, LSV France, LSV Liverpool, LSV TO), Mayfield virus 1 and 2 (MfV1, MfV2), Mill Lade virus (MLV), sacbrood virus (SBV) and SBV‐like virus, slow bee paralysis virus strain Harpenden and Rothamsted (SBPV harp, SBPV roth).

Honey bees and bumble bees showed distinct viromes with only a few viral species overlapping (Figure 2 and Figure S3). Allermuir hill virus (AMV) 1 and 3, Bombus cryptarum densovirus (BCD), MfV2, Mill Lade virus (MLV), and the three putative novel Bt Iflaviruses occurred only in *B. terrestris*, while Apis rhabdo‐like virus 1, all LSV strains, and Bee Macula‐like virus were specific to *A. mellifera* . Most LSVs only occurred locally and at one sampling time point; LSV, LSV1, LSV3 Liaoning, LSV Liverpool, and LSV TO B‐I were only detected in a single library. BQCV (Belle‐Ile and Cherbourg variant), DWV (A and B), slow bee paralysis virus (SBPV Harpenden and Rothamsted variant), SBV, and the SBV‐like virus were found in both species, but except for SBPV Harpenden, those viruses were more abundant and prevalent in *A. mellifera* than in *B. terrestris* . Aphis gossypii virus was also found in both species but only in Quiberon in 2015. MfV1 appeared to be the only virus common in *B. terrestris* that was also found in *A. mellifera* , on the Scilly Isles in 2015, where it accounted for more than 50% of the viral reads. Viromes clustered by host species (Figure 2).

Honey bee viromes were commonly dominated by DWV‐B (> 50% of viral reads in 14 libraries). Both DWV variants were detected in all *A. mellifera* libraries and, on average, accounted for 74% of viral reads (Figure 1 and Figure S1). In comparison, only three *B. terrestris* libraries contained DWV (both variants); in those, DWV‐A and DWV‐B combined accounted for 0.1%– 20% of viral reads. Another common *A. mellifera* virus was the putative novel SBV‐like virus that was found in low abundance (< 0.001%—16% of viral reads) in all *A. mellifera* libraries but also in six *B. terrestris* libraries (0.004%—8% of viral reads). However, only *A. mellifera* from Alderney, Belle‐Ile, and Le Conquet showed high coverage (average depth > 100) for this virus (Table S3). Most *A. mellifera* sample pools (61%) contained at least one and up to three LSV strains. Although, except for some V− islands, including the Scilly Isles in 2021 (81% LSV3 Scilly), Ouessant in 2015 (26% LSV1) and Alderney in 2021 (3% LSV France), LSV strains never accounted for more than 1% of viral reads. The most common variant, named LSV France, was most abundant on the V+ French mainland (Le Conquet, Quiberon, and Cherbourg) but also on the V− island of Alderney and the V+ island of Belle‐Ile. It never co‐occurred with LSV3 Scilly, which, as the name indicates, had a high abundance on the V− Scilly Isles in 2021 and the neighbouring mainland site Penryn in 2015. Other abundant viruses in *A. mellifera* were the two BQCV variants (BQCV Belle‐Ile 52% prevalence, BQCV Cherbourg 48% prevalence, 17% with both variants), SBV (57% prevalence) and the Rothamsted variant of SBPV (43% prevalence). Interestingly, while this SBPV strain clustered with other honey bee‐associated viruses, the SBPV Harpenden strain (only found in *A. mellifera* on Guernsey) clustered with bumble bee viruses (Figure 2). In *B. terrestris* , AMV1, AMV3, MfV1, MLV, BCD, and the putative novel Bt Iflavirus 2 were among the most abundant viruses (prevalence > 45%). The other putative novel Bt Iflavirus 1 was common on Belle‐Ile and its mainland port Quiberon, while Bt Iflavirus 3 only appeared in 2021, where it was most common in Le Conquet and on the Isle of Man (Table S4). Both DWV variants were found in three *B. terrestris* libraries; surprisingly, two of those were from V− islands.

*A. mellifera* libraries had more viral reads (mean = 12.9%, range 0.003%–58%) than *B. terrestris* libraries (mean = 0.7%, range: 0.0008%–3.275%, Wilcoxon *p* < 0.001). This increased proportion of viral reads in *A. mellifera* was driven by V+ mainland (11%) and V+ island (31%) sites, which had a higher proportion of viral reads compared to V− islands (0.1%, Wilcoxon *p* < 0.001 and *p* = 0.001, respectively), with no difference between V+ island and mainland sites (*p* = 0.056). Strikingly, the average DWV‐B read depth in V− sites was below 10^4^ but always more than 10^4^ and on average more than 10^5^ reads in V+ sites (Table S12). In *B. terrestris*, there was no difference between groups (all Wilcoxon *p* > 0.05) and no dominance of one viral species.

### 3.3 Virome Diversity

**FIGURE 3:**
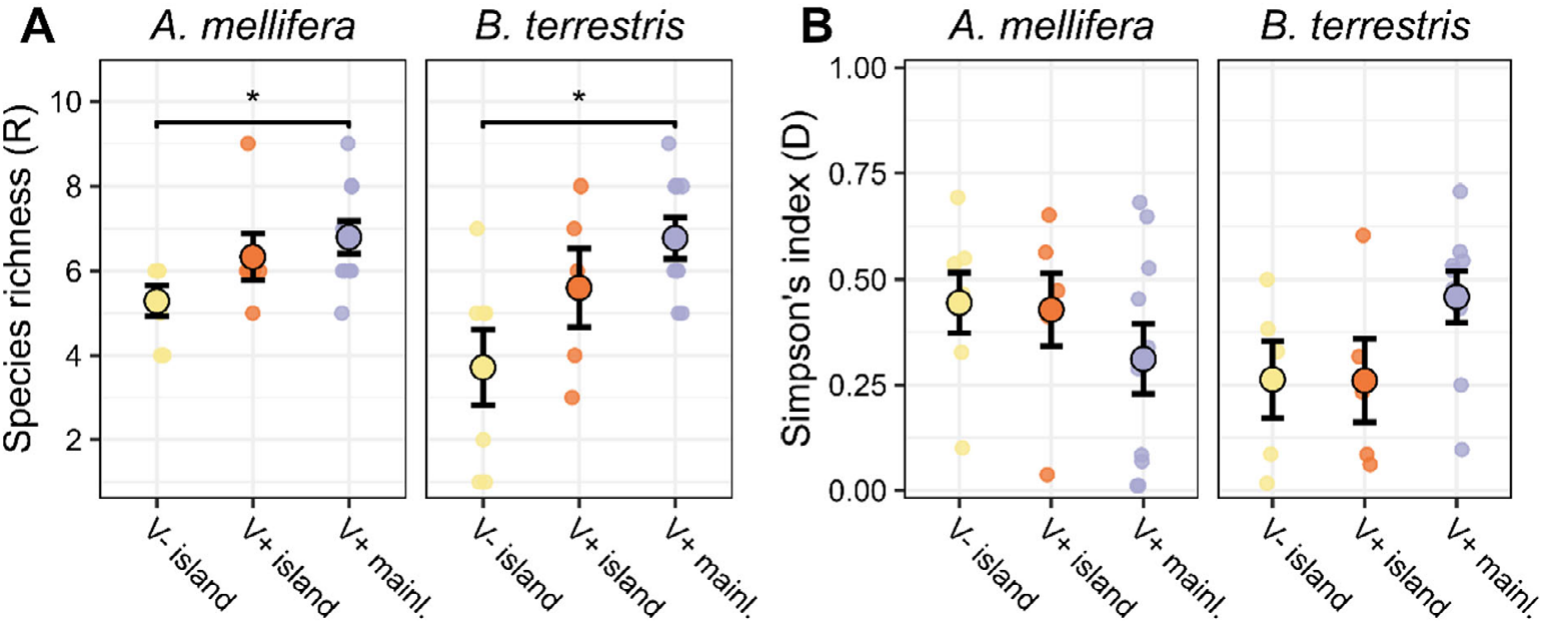
Diversity of *Apis mellifera* and *Bombus terrestris* viromes measured as (A) species richness and (B) Simpson's diversity. Viromes are grouped by varroa‐free islands (V− island, yellow), varroa‐present islands (V+ island, orange), and varroa‐present mainland sites (V+ mainl., purple) and include viromes from both 2015 and 2021. A *t*‐test shows significant differences in richness between V− islands and V+ mainland in both species. * indicates *p* < 0.05.

*A. mellifera* and *B. terrestris* showed no difference in viral species richness (mean richness *R* = 6.22 and *R* = 5.48, respectively, *t*‐test: *p* = 0.200), nor viral alpha diversity (Simpson's D = 0.382 and D = 0.355, respectively, *p* = 0.69). Both species showed similar patterns for viral richness but opposing patterns for viral diversity among groups. In *A. mellifera* , viral species richness was higher on the V+ mainland (*R* = 6.80) than on V− islands (*R* = 5.29, *t*‐test *p* = 0.012), while V+ islands (*R* = 6.33) did not differ from other groups (*p* > 0.05, Figure 3A). Yet, alpha diversity was highest on V− islands (Simpson's D = 0.472), intermediate on V+ islands (D = 0.442) and lowest on the V+ mainland (D = 0.308), although not statistically significant (*p* > 0.05 in pairwise comparison, Figure 3B). In *B. terrestris* , richness was also higher in V+ mainland sites (*R* = 6.78) compared to V− islands (*R* = 3.71, *t*‐test *p* = 0.014) and intermediate on V+ islands (*R* = 5.60, both pairwise tests *p* > 0.05, Figure 3A). In contrast to *A. mellifera* , viral alpha diversity followed this pattern, so that V+ mainland populations were more diverse (D = 0.459) and islands less diverse (V− islands D = 0.263 and V+ islands D = 0.260), although the differences were not significant (*p* > 0.05, Figure 3B). Viral richness can be affected by the number of individuals in the sampling pool, but differences between V− islands and V+ mainland sites in both host species remained when excluding the three pools with fewer than 28 individuals ( *A. mellifera* *p* = 0.045 and *B. terrestris* *p* = 0.040).

**FIGURE 4:**
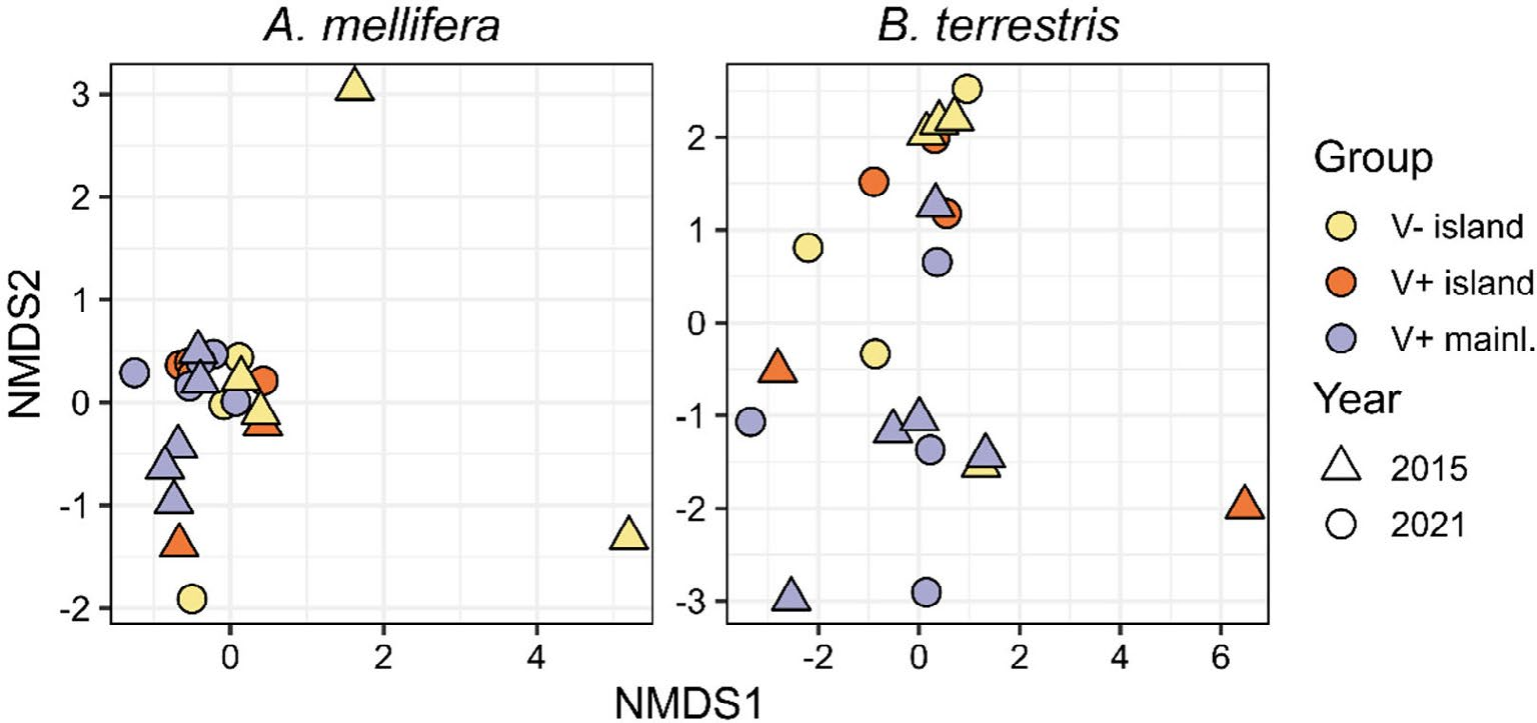
Non‐metric Multi‐Dimensional Scaling (NMDS) ordination plots, based on Bray–Curtis dissimilarity matrices of *Apis mellifera* (left) and *Bombus terrestris* (right) viromes. Stress in *A. mellifera* MDS = 0.057 and in *B. terrestris* MDS = 0.168. Shapes indicate the sampling year (triangles 2015 and circles 2021) and colours whether bees were collected on a varroa‐free (yellow) or varroa‐present (orange) island (V− or V+ island) or the varroa‐present mainland (V+ mainl., purple).

The virus species composition in *A. mellifera* differed with varroa presence (PERMANOVA: Pseudo‐*F* = 4.651, *p* = 0.001, *R* ^2^ = 0.158) but also shifted over time (Pseudo‐*F* = 2.161, *p* = 0.048, *R* ^2^ = 0.073, Figure 4 and S4), while island location or geography (latitude) did not affect community composition (both *p* > 0.05). The three V− islands that clustered furthest from all other samples were the Scilly Isles and Ouessant in 2015 and the Scilly Isles in 2021 (in a clockwise order, Figure 4). The temporal shift was driven by a shift in the DWV variant. When we combined DWV‐A and DWV‐B reads, the temporal signal disappeared (*F* = 1.185, *p* = 0.317, *R* ^2^ = 0.043) and the varroa effect remained (*F* = 3.905, *p* = 0.001, *R* ^2^ = 0.141). The viral species composition in *B. terrestris* was structured by latitude (PERMANOVA: *F* = 3.524, *p* = 0.001, *R* ^2^ = 0.144) but not by sampling year, island location, or varroa presence (all *p* > 0.05, Figure 4 and Figure S4).

### 3.4 Temporal Shifts in DWV Sequences

**FIGURE 5:**
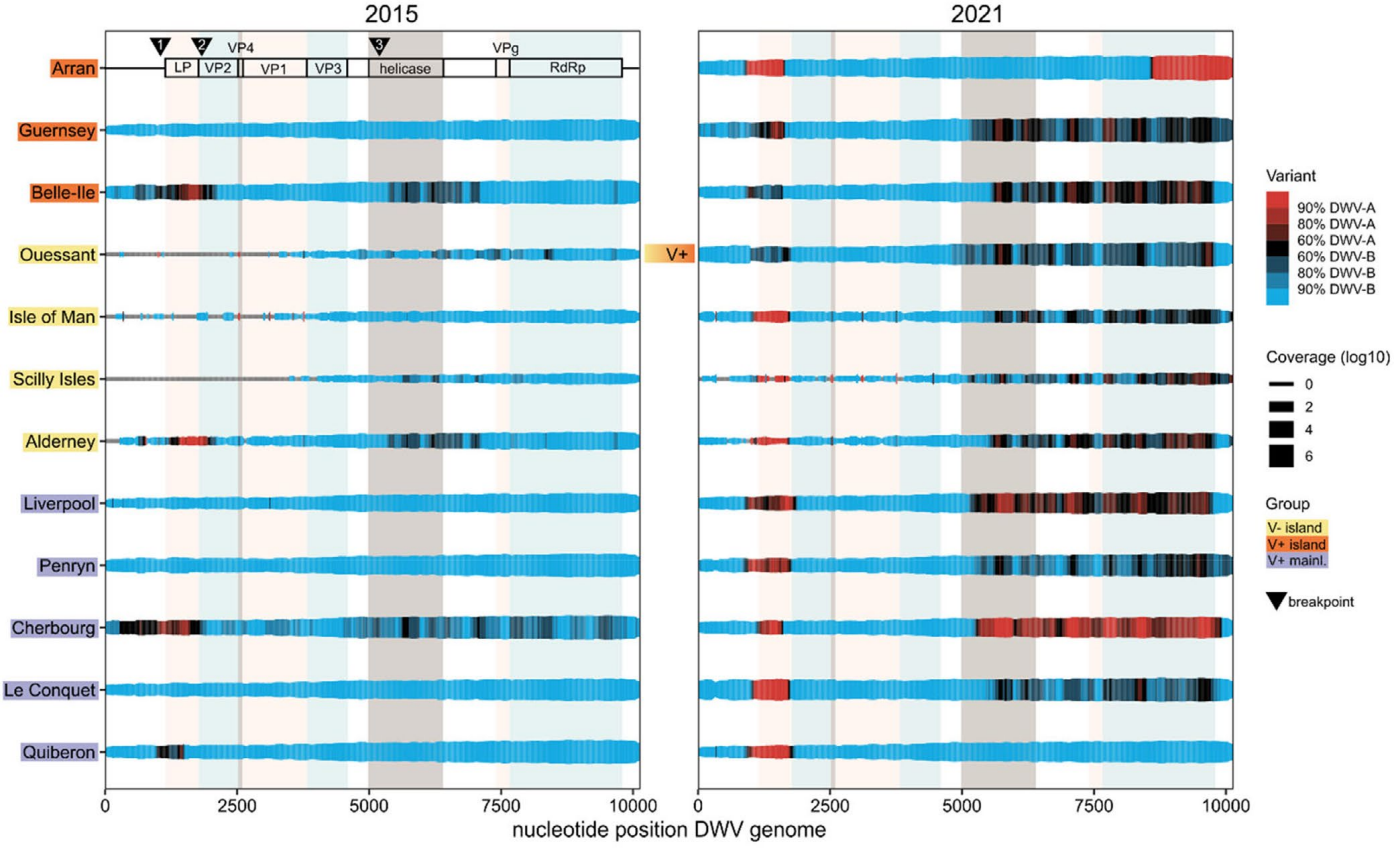
Deformed wing virus read coverage and variants in *Apis mellifera* viral communities in 2015 and 2021. The colours of the lines show the ratio of DWV‐A to DWV‐B reads at each nucleotide position in the DWV genome, with red colours indicating a majority of DWV‐A reads, blue for a majority of DWV‐B reads, and black for similar amounts of both variants. The line width indicates the log10 transformed coverage; note that 0 coverage towards the 5′ end is presented in grey. The left upper panel shows a map of the DWV genome: 5′UTR, *leader protein* (*LP*) gene, *viral capsid protein* genes *VP2*, *VP4*, *VP1*, *VP3*, and the non‐structural protein genes with the *helicase*, *VPg*, and *RNA‐dependent RNA‐polymerase* (*RdRp*) genes and the approximate positions of the three breakpoints confirmed by Sanger sequencing are indicated as triangles. The breakpoints potentially separate the genome into four blocks that result in recombinants with a BABB or BABA genome, mostly in 2021. The coloured stripes in the background provide a reference to the genomic features. Sites are arranged by varroa‐present (V+) islands (orange highlighting), varroa‐free (V−) islands (yellow), and varroa‐present (V+) mainland sites (purple), within each group sorted from north to south.

Counter to the expectation of an increase in DWV‐B reads in more recent samples, we find that DWV‐A reads increased from 2015 to 2021 (Figure 1). The DWV genome coverage shows that in 2015 most *A. mellifera* libraries had a majority of DWV‐B reads, except for some sites with higher DWV‐A than DWV‐B coverage in the *leader protein* (*LP*) gene in France (Cherbourg, Belle‐Ile, Quiberon, and the nearby Alderney). Yet by 2021, DWV‐A coverage in the *LP* gene increased and DWV‐B coverage decreased so that most libraries had higher DWV‐A than DWV‐B coverage in this region (Figure 5, Figures S5 and S6). Additionally, some sites (particularly Cherbourg and Liverpool) also showed a higher coverage for DWV‐A than DWV‐B reads in genes encoding the non‐structural proteins (Figure 5). The 5′ untranslated region (UTR) and structural proteins had higher DWV‐B than DWV‐A coverage in all sites and both years. It is worth noting that although the dominant variant changed, all *A. mellifera* libraries had > 93% coverage of the *RdRp* gene for both DWV variants in both years (Figures S5 and S6). Low genome coverage towards the 5′ end could be linked to lower infection load (in V− sites) and poly(A) selection during library preparation, but 'gaps' in one variant's coverage with high coverage of the other may indicate the occurrence of recombinant strains. Although bias during random priming, reverse transcription or other processing steps that can result in variant coverage not always presenting the true nature of the sample cannot be excluded as causes for the observed pattern, these shifts in DWV variants and potential occurrence of recombinant strains appeared independent of island or mainland location and showed no link to varroa‐presence (Figure 5). And although average read depth in *B. terrestris* was much lower (< 10^2^), coverage plots indicated the presence of similar strains in the three positive bumble bee libraries (Figure S7).

Assembled contigs suggested five potential breakpoints in the DWV genome (Figure 5): a first switch in the 5′ UTR shortly before the ORF from DWV‐B to DWV‐A at 843–1162 bases, a second with a switch from DWV‐A to DWV‐B at the start of the *VP2* gene between 1621 and 1706 bases, a third after the structural proteins at the start of the *helicase* gene from DWV‐B to DWV‐A from 5266‐5536 bases, a fourth at 6225–6250 bases (end of helicase) and a fifth at 7712–7783 bases (start of *RdRp* gene), although break‐point four and five could not be confirmed using Sanger sequencing and are not shown in Figure 5. The first breakpoint from B to A shortly before the ORF was found in almost all V+ island and mainland sites in 2021 (Arran, Liverpool, Penryn, Cherbourg, Guernsey, Le Conquet, Ouessant, and Quiberon) through amplification of the breakpoint region and Sanger sequencing (details in supplemental material S3). The second switch from A to B at the start of the *VP2* gene was only found in Sanger sequences from Arran and Quiberon, although genome coverage plots show a switch back to DWV‐B in the *VP2* gene in all 2021 libraries. The third breakpoint after the *VP* genes, at the beginning of the non‐structural proteins, was found in sequences from Liverpool, Cherbourg, and Guernsey, even though other libraries had similarly high ratios of DWV‐A reads for this part of the genome. Although we were unable to amplify breakpoint positions for V− islands, likely due to the lower DWV coverage, genome coverage suggests the presence of similar recombinant strains. Taken together, a variant with a DWV‐B 5′ UTR, a DWV‐A LP, DWV‐B VPs, and DWV‐A or DWV‐B non‐structural proteins (BABA or BABB) may be common in 2021. These variants may co‐occur with the pure DWV‐A and DWV‐B variants, although we only retrieved full coverage for pure DWV‐A from Cherbourg in 2015 and Guernsey in 2021 (< 87% in other libraries, mean coverage 61%, Figure S5), while all V+ *A. mellifera* populations had 100% DWV‐B coverage in 2015 and more than 94% coverage of DWV‐B in 2021 (Figure S7).

Using PCR prevalence screening of individuals that were pooled for RNA‐seq showed that co‐occurrence of multiple DWV types was not only caused by pooling bees, but that instead 96% of DWV‐A positive individuals also carried DWV‐B, while only 37% of DWV‐B positives in 2015 and 21% in 2021, carried the other variant (Table S12). Deep sequencing was more sensitive than PCR and detected DWV on multiple V− islands for which no PCR‐positives were detected. Those were *A. mellifera* on Alderney (2015), the Isle of Man (2021) and the Scilly Isles (both years), and *B. terrestris* on Alderney (2015) and the Scilly‐Isles (2021). Yet, in some *B. terrestris* pools, PCR indicated DWV presence, but only fragments of the RdRp (12%–73%) were covered by RNA‐seq reads, not meeting our criteria for detection (Table S12).

## 4 Discussion

In this study, we show that ecological and evolutionary factors shape the richness, diversity and composition of viromes in honey bees and bumble bees. Wild *B. terrestris* showed geographical structure in viral species composition and the decrease in viral richness predicted by the theory of island biogeography. While managed *A. mellifera* had lower viral richness on islands, their viromes were affected by the presence of the virus‐vectoring varroa mite and indicated a temporal shift in emerging DWV variants. The presence of varroa was linked to the dominance of DWV, which appeared to have shifted from mostly DWV‐B in 2015 to a mix of pure DWV‐B and recombinant strains in 2021. The global emergence of DWV‐B and replacement of DWV‐A (Paxton et al. 2022) may be followed by the rise of recombinant strains, as observed here but also in other European populations and in the US (Hesketh‐Best et al. 2024; Sircoulomb et al. 2025).

Multiple independently evolving functional blocks in the DWV genome may allow exchange between closely related strains (Dalmon et al. 2017; Moore et al. 2011). Recombination can increase the adaptive rate and mitigate the effects of a small population size and high mutation rate (Xiao et al. 2016) by combining co‐circulating adaptive mutations and removing deleterious mutations (Simon‐Loriere and Holmes 2011). Here, we have used targeted PCR and Sanger sequencing to support evidence of potential recombinant DWV strains, previously also reported from short‐read (Dalmon et al. 2017; Moore et al. 2011) and also long‐read sequencing (Hesketh‐Best et al. 2024; Sircoulomb et al. 2025). Our results suggest that the three described functional blocks of the 5′ UTR, the leader and capsid protein‐encoding regions and the region encoding the non‐structural proteins (Moore et al. 2011) may harbour an additional breakpoint separating the leader and capsid‐encoding region, resulting in four blocks. Multiple European populations report variants with a DWV‐A *LP* and DWV‐B *VP* similar to the BABA and BABB variants that we find in pooled samples from 2021. These variants either have a DWV‐B 5′ UTR, such as in the Netherlands (BABB), France (BABA/BABB), Spain (BABA), and the UK (BABB) (Dalmon et al. 2017; Mordecai, Brettell, et al. 2016; Norton et al. 2020; Sircoulomb et al. 2025) or a DWV‐A 5′ UTR in the UK (AABA or AABB) (Cornman 2017; Ryabov et al. 2014; Wang et al. 2013) and non‐structural proteins from either variant. Recombination between the structural and non‐structural protein encoding regions has been found outside of Europe but seems less common within the *VP2* genes or the 5′ UTR (Cornman 2017; Hesketh‐Best et al. 2024; Şevik et al. 2024; Zioni et al. 2011).

Although DWV‐A and DWV‐B both actively infect varroa mites (Damayo et al. 2023), it is not clear how varroa interacts with DWV or why the mites' immune reaction differs from that to a varroa‐associated pathogen (Eliash, Suenaga, and Mikheyev et al. 2022). Interestingly, the rare DWV‐C was recently found at high prevalence on two varroa‐free islands in the Azores archipelago (Lopes et al. 2024). The increased competitive advantage of DWV‐B over DWV‐A that was linked to the decrease of DWV‐A appears to be independent of varroa (Norton et al. 2020). Yet, how DWV‐C or recombinants between A and B compare to the wild types has not been tested yet. Varroa presence was not linked to the appearance of recombinant strains in this study. Coverage of DWV in V− islands of Alderney, the Isle of Man, and the Scilly Isles here and varroa‐free Åland in Finland (Sircoulomb et al. 2025) all suggest the occurrence of recombinant strains. Although co‐infections of DWV‐A and DWV‐B that provide opportunities for recombination should be rare in varroa‐free populations that have lower DWV prevalence. Sample pools only show the virome at a population level and not within individuals, where recombination events occur. Yet, PCR testing showed that 96% of individuals with a type A DWV in our pools were also infected with DWV‐B, and co‐infections with multiple DWV strains appear common in beehives (Kevill et al. 2019; Ryabov et al. 2017). Whether recombinant variants arose independently on V− islands or whether they spread from V+ populations is not clear. Few sites in 2015 showed DWV‐A coverage across the *LP*, although its coverage was high in 2021, suggesting that DWV‐A in this functional block could have been rare in 2015 so that we did not detect it in our pools of 30 bees or that it was introduced from elsewhere. The presence of DWV‐B on V− islands in Europe suggested that DWV can travel ahead of its vector (Manley et al. 2019), so that phylogenetic analyses of functional blocks may reveal the recombinant origin.

As transmission routes are key in viral dynamics (Richard and Fouchier 2015), varroa and the vector‐mediated transmission it introduced affected viral community composition in *A. mellifera* . Despite V+ mainland sites harbouring the highest species richness, their diversity was lowest due to uneven viral abundances and high DWV abundance. Varroa itself can also be dominated by DWV reads: A recent study from China revealed that although varroa hosts a rich virome, DWV accounted for over 90% of reads, more than double the amount found in *A. mellifera* and more than ten times the amount in *A. cerana* (Li et al. 2023). Yet, the effects of vector transmission on bee viruses differ depending on the virus. The diverse group of LSV strains, which we find in all sites except the Isle of Man, shows higher richness in varroa‐present compared to varroa‐free islands in the Azores (Lopes et al. 2024), and increased LSV2 titres have been linked to varroa presence (Doublet et al. 2024). BQCV and SBV that were present in most libraries likely become too virulent when transmitted by a vector, while the relatively low virulence of DWV allows for pupal survival and adult emergence (al Naggar and Paxton 2020; McMahon et al. 2018; Remnant et al. 2019) and may lead to the dominance of DWV in varroa‐infested populations. The high proportion of viral reads in *A. mellifera* compared to *B. terrestris* aligns with other comparisons to wild bees (Doublet et al. 2025; Robinson et al. 2024) and is likely linked to varroa increasing viral titres (Manley et al. 2019; Mondet et al. 2014), as viruses only accounted for 0.1% of reads in varroa‐free honey bee libraries. This indicates that, similar to wild bees, varroa‐free honey bees have a relatively even distribution of lowly abundant viruses.

DWV and other honey bee‐associated viruses were rare in co‐foraging *B. terrestris* , and bumble bees were not affected by varroa presence. This contrasts with PCR and qPCR‐based findings of increased prevalences of honey bee‐associated viruses in bumble bees at sites with varroa (Manley et al. 2020, 2019) and did not always match the PCR detection of DWV in individual bumble bees that were included in the pooled samples. Yet, as we only considered viruses for which more than 33% of the genome and at least 75% of the RdRp were covered, lowly abundant viruses were filtered out. This suggests that PCR detection of DWV RNA in bumble bees may arise from environmental sources (Gusachenko et al. 2020) such as contaminated pollen (Mazzei et al. 2014) and could present degraded RNA fragments and not an active infection. Overall, honey bee‐associated viruses were rare in *B. terrestris* viromes, indicating that viral spill‐over and virus‐virus interactions arising from spill‐over may not affect the virus community (but see Manley et al. 2019). Instead, wild bumble bees have distinct viromes (Doublet et al. 2025; Pascall et al. 2021; Robinson et al. 2024; Schoonvaere et al. 2018) that showed different community composition with latitude and in which we discovered three putative novel viruses. No virus was shared across all *B. terrestris* libraries, and as in *A. mellifera* , island populations had lower species richness compared to the mainland.

Species mobility and with it virus distribution can be affected by geographic barriers; for example, bees rarely cross large water bodies such as those surrounding island populations (Estoup et al. 1996; Fijen 2021; Goulson et al. 2011; Widmer et al. 1998). Infectious diseases remain constrained in their distributions by ecological barriers (Murray et al. 2015), but human‐mediated movement can erode such barriers. We assumed that honey bees have greater mobility than wild bumble bees due to trade. Although it should be noted that bumble bees, particularly *B. terrestris*, are increasingly traded for pollination in greenhouses. Indeed, viral species richness in wild and managed bees followed the predictions of island biogeography, with fewer species in smaller and more isolated island habitats (MacArthur and Wilson 1967). This pattern has also been shown for amphibian viruses, which have lower diversity on islands compared to mainland sites (Wang et al. 2017) and avian blood parasites, which decrease in richness with distance to the mainland (Pérez‐Rodríguez et al. 2013).

While differences in virome composition between species can be attributed to differences in the biogeographic and evolutionary history of hosts (French et al. 2024; Mahar et al. 2024) specifically also in bees (Doublet et al. 2025; Pascall et al. 2019), the more recent focus on variation within species has revealed a diverse range of factors that shape communities. Seasonal variation (Feng et al. 2022; Raghwani et al. 2023), population age structure (Bergner et al. 2020), interactions with co‐infecting viruses (Pascall et al. 2021; Wille et al. 2018), or the order in which infections are acquired (Jokinen et al. 2023) all affect virome composition across a range of taxa and highlight the need to include multiple populations and time points to understand the factors shaping bee viromes. Yet, sample pools used here cannot give insights into processes in individual bees that ultimately shape community composition and may also reduce sensitivity for rare or lowly abundant viruses.

Our results demonstrate that virus richness in bees follows the predictions of island biogeography theory, with fewer species on islands. Species' mobility further affected the virome. In wild bumble bees, geographic location had the strongest impact on virome composition, while in managed honey bees, virome composition was similar across large geographic scales. The rare insight into varroa‐naïve honey bee viromes further revealed a diverse RNA virus community that includes DWV and its emerging recombinant strains without being dominated by this virus. Human trade and the invasion of varroa have severely affected the honey bee virome. Using an ecological framework to study virome composition can provide valuable insight into the barriers of disease spread and could help to predict drivers of disease emergence.

## Author Contributions

J.D. and L.W. designed and performed the research. J.D. analysed the data and wrote the manuscript with contributions from L.W.

## Conflicts of Interest

The authors declare no conflicts of interest.

## Supporting information


**Data S1:** Supporting information.

## Data Availability

RNA‐seq reads from this study have been deposited in the NCBI Sequence Read Archive under SRA project accession PRJNA1233015. Assembled virus genomes are available on GenBank under accession numbers PV239920 to PV239933. The raw data and analysis scripts supporting the findings of this study are publicly available on Dryad (DOI: https://doi.org/10.5061/dryad.jsxksn0ns).
