# Supplementary material for: Bumble Bees and Honey Bees on Islands Harbour Reduced Viral Species Richness, Yet Honey Bee Populations Are Dominated by a Deformed Wing Virus Recombinant
Source: Mol Ecol. 2025 Aug 11;34(24):e70070. doi: 10.1111/mec.70070 (PMC12717974; doi:10.1111/mec.70070)
Supplement: Supplementary file 1 — Data S1: Supporting information. [file MEC-34-e70070-s001.docx]

Supplemental material

Bumble bees and honey bees on islands harbour reduced viral species richness, yet honey bee populations are dominated by a deformed wing virus recombinant

Jana Dobelmann^1^ & Lena Wilfert^1^

^1^ Institute of Evolutionary Ecology and Conservation Genomics, University of Ulm, Albert-Einstein-Allee 11, 89081 Ulm, Germany

## Methods

S1: PCR to distinguish *Bombus* species

We used a PCR with a species-specific length polymorphism that amplifies part of the *cytocrome oxidase subinut 1* (CO1) gene to distinguish between *Bombus terrestris* and *B. lucorum*. We extracted DNA from a single hind leg using 200 µl Chelex (5% wt/vol) and 20 µl proteinase K (100 µM, Roche). Incubation was at 56 °C for 1 h with shaking and inactivation at 96 °C for 15 min. Extracts were centrifuged for 1 min at 21,300 *g* and 150 µl supernatant was removed to recover DNA. 1 µl 1:5 diluted DNA was used in a 15 µl reaction with 2.5 mM MgCl_2,_ 0.2 mM dNTPs, 0.5 µM primer each (F: GGAGCAATAATTTCAATAAATAG and R: AARTTCAAAGCACTAATCTGC) and 0.375 U GoTaq® Flexi DNA Polymerase (Promega). Cycling was 5 min at 94°C, followed by 35 cycles of 15 sec 94°C, 20 s 55°C and 20 s 72°C, followed by 5 min 72°C. Amplicons were visualised on a 1.5% agarose gel with RedSafe gel stain (Intron Biotechnology) and showed a 350 bp fragment for *B. terrestris* and 286 bp for *B. lucorum*. In 2015, 38 *B. lucorum* and in 2021, 28 *B. lucorum* were detected and excluded (Dobelmann & Wilfert, 2024; Manley et al., 2019). The *B. lucorum* in 2021 were found on Arran (13), Ouessant (10), the Isle of Man (3), Belle-Ile (1) and Penryn (1).

S2: Reference sequence alignments

The nucleotide reference library used for read mapping contained 46 viral references (Table S6). To ensure that this reference database contained distinct viral sequences that would allow for specific read mapping, we used pairwise comparisons of RNA dependent RNA polymerase (RdRP) identity within families. RdRp annotations were retrieved for reference sequences downloaded from GenBank ([www.ncbi.nlm.nih.gov/genbank/](http://www.ncbi.nlm.nih.gov/genbank/)) or predicted using the “predict annotation” function in Geneious Prime v2025.1.2 (Kearse et al., 2012) using annotated RdRps from RdRp-scan (Charon et al., 2022) downloaded on 9.01.23 (<https://github.com/JustineCharon/RdRp-scan/blob/main/RdRp-scan_0.90.fasta>) and selected the closest RdRp match. Viruses were grouped into families (if known), RdRp regions extracted and aligned using Muscle 5.1 Super5 (Edgar, 2004), trimmed if necessary and similarities calculated. RdRp identities between viruses can be found in Tables S7 – S11.

S3: Screening individual bees for DWV-A and DWV-B

600-3750 ng RNA from individual bees used in RNA-seq pools for 2021 was reverse transcribed using random hexamers and GoScript™ reverse transcriptase (Promega) according to the manufacturer's instructions to screen for the *viral capsid protein* genes of deformed wing virus type A and B (DWV-A and DWV-B). PCR was performed using the GoTaq® Flexi (Promega) kit using 0.375 U GoTaq® Flexi DNA Polymerase in a 15 µl reaction with F: AAATCCGGTATGGGAAGTCATGC and R: CTGCCTGAGCTTCTCTAACTTCG for DWV-A (929 bp fragment) and F: GAGATATGGAAGTTCGAGTGCAGA and R: TTGCCCTAATTTGATTCGCGTAAA for DWV-B (405 bp fragment) (Dobelmann et al., 2024). Cycling for DWV-A was 94°C for 2 min, 38 cycles of 20 s at 94°C, 30 s at 55°C, 45s 72°C, and a final 5 min at 72°C. The cycling protocol for DWV-B was 94°C for 2 min, 35 cycles of 20 s at 94°C, 30 s at 60°C, 30 s at 72°C, followed by a final 5 min at 72°C. Every run included a known virus-positive sample and water as a negative control. 5 µl PCR product were visualised by 1.5% TAE agarose gel electrophoresis and staining with RedSafe^TM^ (Intron Biotechnology) using HyperLadder™ 50 bp (Bioline) to check the fragment size. Prevalence data for 2015 samples was retrieved from Manley et al. (2019).

## Tables

Table S1:Locations and sampling time points for *Apis mellifera* and *Bombus terrestris* collections in 2015 and 2021. Samples in 2015 were collected by of Manley et al. (2019). Due to travel restriction in 2021, Cherbourg was collected later in the season. * *A. mellifera* only.

| Group | Site | Latitude | Longitude | Collection 2015 | Collection 2021 |
| --- | --- | --- | --- | --- | --- |
| V- island | Alderney | 49.71321 | -2.20692 | 16. -17. July | 22. – 24. August |
|  | Scilly Isles | 49.91428 | -6.30417 | 23. and 25. July | 20. July |
|  | Isle of Man (Douglas) | 54.15243 | -4.48584 | 29. – 30. July | 19. - 23. July |
| V+ mainland | Quiberon | 47.4822 | -3.12019 | 24. – 25. June | 21. June |
|  | Le Conquet | 48.35945 | -4.77332 | 20. – 23. June | 22. and 26. June |
|  | Cherbourg | 49.65157 | -1.62054 | 18. July | 20. -21. September* |
|  | Penryn | 50.15276 | -5.06717 | 5. – 6. July | 19. – 23. July |
|  | Liverpool | 53.40759 | -2.99091 | 31. July | 19. – 23. July |
| V+ island | Belle-Ile (Le Palais) | 47.34712 | -3.15538 | 26. June | 15. – 16. June |
|  | Ouessant | 48.45746 | -5.09644 | 21. – 23. June | 22. -24. June |
|  | Guernsey (St Peter Port) | 49.45977 | -2.53468 | 10. and 15. July | 21. – 25. August |
|  | Arran (Brodick) | 55.59502 | -5.15081 | - | 26. August* |

Table S2: Data generated from RNA-Seq libraries in this study that can be found in the NCBI sequences read archive under BioProject PRJNA1233015 . N shows the number of bees pooled.

| ID | Name | Site | Year | Species | N | no. of reads | data yield [GB] |
| --- | --- | --- | --- | --- | --- | --- | --- |
| 1 | Arran Am 21 | Arran | 2021 | *A. mel* | 30 | 219,048,216 | 27.1 |
| 2 | Alderney Am 21 | Alderney | 2021 | *A. mel* | 28 | 210,643,014 | 26.7 |
| 3 | Belle-Ile Am 21 | Belle-Ile | 2021 | *A. mel* | 30 | 235,753,542 | 31.4 |
| 4 | Cherbourg Am 21 | Cherbourg | 2021 | *A. mel* | 30 | 218,928,670 | 28.4 |
| 5 | Guernsey Am 21 | Guernsey | 2021 | *A. mel* | 30 | 208,164,726 | 25.9 |
| 6 | IOM Am 21 | Isle of Man | 2021 | *A. mel* | 30 | 236,524,912 | 31.4 |
| 7 | Le Conc. Am 21 | Le Conquet | 2021 | *A. mel* | 30 | 212,957,528 | 28.3 |
| 8 | Liverpool Am 21 | Liverpool | 2021 | *A. mel* | 30 | 216,477,176 | 28.7 |
| 9 | Oues Am 21 | Ouessant | 2021 | *A. mel* | 30 | 237,094,020 | 31.5 |
| 10 | Penryn Am 21 | Penryn | 2021 | *A. mel* | 30 | 235,392,248 | 31.3 |
| 11 | Quiberon Am 21 | Quiberon | 2021 | *A. mel* | 30 | 233,808,220 | 30.8 |
| 12 | Scilly Am 21 | Scilly Isles | 2021 | *A. mel* | 30 | 234,035,830 | 31.4 |
| 13 | Alderney Bt 21 | Alderney | 2021 | *B. ter* | 30 | 236,114,234 | 31.5 |
| 14 | Belle-Ile Bt 21 | Belle-Ile | 2021 | *B. ter* | 30 | 234,359,230 | 31.2 |
| 15 | Guernsey Bt 21 | Guernsey | 2021 | *B. ter* | 17 | 227,731,968 | 31.4 |
| 16 | IOM Bt 21 | Isle of Man | 2021 | *B. ter* | 30 | 208,681,608 | 29.4 |
| 17 | Le Conquet Bt 21 | Le Conquet | 2021 | *B. ter* | 30 | 234,581,384 | 32.0 |
| 18 | Liverpool Bt 21 | Liverpool | 2021 | *B. ter* | 30 | 235,446,266 | 32.0 |
| 19 | Oues Bt 21 | Ouessant | 2021 | *B. ter* | 30 | 236,918,022 | 32.3 |
| 20 | Penryn Bt 21 | Penryn | 2021 | *B. ter* | 30 | 236,325,658 | 31.9 |
| 21 | Quiberon Bt 21 | Quiberon | 2021 | *B. ter* | 30 | 237,684,514 | 30.9 |
| 22 | Scilly Bt 21 | Scilly Isles | 2021 | *B. ter* | 30 | 237,387,074 | 30.4 |
| 23 | Alderney Am 15 | Alderney | 2015 | *A. mel* | 30 | 230,163,910 | 30.2 |
| 24 | Belle-Ile Am 15 | Belle-Ile | 2015 | *A. mel* | 28 | 236,964,368 | 32.0 |
| 25 | Cherbourg Am 15 | Cherbourg | 2015 | *A. mel* | 30 | 236,762,330 | 30.8 |
| 26 | Guernsey Am 15 | Guernsey | 2015 | *A. mel* | 22 | 237,523,728 | 31.2 |
| 27 | IOM Am 15 | Isle of Man | 2015 | *A. mel* | 30 | 235,707,394 | 31.4 |
| 28 | Le Conquet Am 15 | Le Conquet | 2015 | *A. mel* | 30 | 237,450,710 | 32.9 |
| 29 | Liverpool Am 15 | Liverpool | 2015 | *A. mel* | 29 | 236,258,838 | 31.3 |
| 30 | Oues Am 15 | Ouessant | 2015 | *A. mel* | 30 | 234,082,370 | 30.8 |
| 31 | Penryn Am 15 | Penryn | 2015 | *A. mel* | 30 | 213,746,790 | 30.1 |
| 32 | Quiberon Am 15 | Quiberon | 2015 | *A. mel* | 30 | 235,719,762 | 30.5 |
| 33 | Scilly Am 15 | Scilly Isles | 2015 | *A. mel* | 30 | 235,719,762 | 28.8 |
| 34 | Alderney Bt 15 | Alderney | 2015 | *B. ter* | 30 | 236,028,932 | 29.9 |
| 35 | Belle-Ile Bt 15 | Belle-Ile | 2015 | *B. ter* | 30 | 235,248,048 | 29.3 |
| 36 | Cherbourg Bt 15 | Cherbourg | 2015 | *B. ter* | 30 | 235,399,084 | 29.5 |
| 37 | Guernsey Bt 15 | Guernsey | 2015 | *B. ter* | 30 | 235,913,804 | 30.2 |
| 38 | IOM Bt 15 | Isle of Man | 2015 | *B. ter* | 30 | 236,299,788 | 29.0 |
| 39 | Le Conquet Bt 15 | Le Conquet | 2015 | *B. ter* | 30 | 227,747,442 | 29.7 |
| 40 | Liverpool Bt 15 | Liverpool | 2015 | *B. ter* | 30 | 215,343,328 | 26.6 |
| 41 | Oues Bt 15 | Ouessant | 2015 | *B. ter* | 13 | 227,014,742 | 27.9 |
| 42 | Penryn Bt 15 | Penryn | 2015 | *B. ter* | 30 | 235,795,766 | 29.8 |
| 43 | Quiberon Bt 15 | Quiberon | 2015 | *B. ter* | 30 | 235,870,568 | 29.1 |
| 44 | Scilly Bt 15 | Scilly Isles | 2015 | *B. ter* | 30 | 236,683,230 | 30.6 |

Table S3: Nucleotide reference database used for mapping reads to determine presence (>75% RdRp coverage and >33% genome coverage) and abundance of viruses in bee metatrascriptomes. *For the only DNA virus (Bombus cryptarum densovirus), >33% coverage in the structural protein 1 was used instead of the RdRp. References included were identified through blastx searches of *de novo* assembled contigs against a large protein database supplemented by 6 known bee viruses. Those included: ABPV, ALPV, DWV-C, DWV-D, IAPV and KBV, which were not detected. Detected viruses (column D) are indicated by “Y” and viruses that were not detected as “N”. Note that the genome organization for unclassified Riboviria is unknown. Accession numbers highlighted in bold were generated by *de novo* assembly in this study.

| Virus | Abbrev. | GenBank Accession | Family | Genome | Genome | RdRp | | D |
| --- | --- | --- | --- | --- | --- | --- | --- | --- |
|  |  |  |  |  |  | Start | End |  |
| Acute bee paralysis virus | ABPV | NC_002548 | Dicistroviridae | ssRNA(+) | 9470 | 4877 | 6322 | N |
| Allermuir Hill virus 1 | AMV1 | MH614288 | uncl. Riboviria | NA | 7586 | 3763 | 5187 | Y |
| Allermuir Hill virus 2 | AMV2 | MH614289 | uncl. Riboviria | NA | 9078 | 6391 | 7809 | N |
| Allermuir Hill virus 3 | AMV3 | MH614290 | uncl. Riboviria | NA | 6339 | 2512 | 3930 | Y |
| Aphid lethal paralysis virus | ALPV | NC_004365 | Dicistroviridae | ssRNA(+) | 9812 | 4875 | 6644 | N |
| Aphis gossypii virus | AGV | MH476203 | Dicistroviridae | ssRNA(+) | 102004 | 4930 | 6686 | Y |
| Apis rhabdovirus 1 | ARV1 | MT636362 | Rhabdoviridae | ssRNA(-) | 14583 | 8169 | 9962 | Y |
| Apis rhabdovirus 2 | ARV2 | MZ821796 | Rhabdoviridae | ssRNA(-) | 14001 | 7015 | 8808 | N |
| Apis rhabdovirus 5 | ARV5 | MZ822108 | Rhabdoviridae | ssRNA(-) | 13405 | 8033 | 9820 | N |
| Bee Macula-like virus | BMlV | NC_027631 | Tymoviridae | ssRNA(+) | 6244 | 3905 | 5449 | Y |
| Black queen cell virus Cherbourg variant | BQCV Cherb. | **PV239921** | Dicistroviridae | ssRNA(+) | 8429 | 3735 | 5501 | Y |
| Black queen cell virus Belle-Ile variant | BQCV B-I | **PV239920** | Dicistroviridae | ssRNA(+) | 8436 | 3743 | 5509 | Y |
| Boghill Burn virus | BBV | MH614292 | uncl. Riboviria | NA | 9873 | 5084 | 6241 | N |
| Bombus cryptarum densovirus | BCD | NC_040626 | Parvoviridae | ssDNA | 3977 | *238** | *1068** | Y |
| Bt Iflavirus 1 | Bt Iflavirus 1 | **PV239922** | Iflaviridae | ssRNA(+) | 9233 | 7366 | 9150 | Y |
| Bt Iflavirus 2 | Bt Iflavirus 2 | **PV239923** | Iflaviridae | ssRNA(+) | 9229 | 7370 | 9160 | Y |
| Bt Iflavirus 3 | Bt Iflavirus 3 | **PV239924** | Iflaviridae | ssRNA(+) | 8927 | 7042 | 8799 | Y |
| Deformed wing virus A | DWV-A | **PV239925** | Iflaviridae | ssRNA(+) | 10141 | 8157 | 9830 | Y |
| Deformed wing virus B | DWV-B | **PV239926** | Iflaviridae | ssRNA(+) | 10127 | 8144 | 9817 | Y |
| Deformed wing virus C | DWV-C | CEND01000001 | Iflaviridae | ssRNA(+) | 10123 | 8139 | 9812 | N |
| Deformed wing virus D | DWV-D | MT504363 | Iflaviridae | ssRNA(+) | 10120 | 8147 | 9820 | N |
| Dumyat virus | DV | MH614299 | uncl. Riboviria | NA | 4981 | 3036 | 4826 | N |
| Gorebridge virus | GV | MH614301 | uncl. Riboviria | NA | 5639 | 901 | 2550 | N |
| Israel acute paralysis virus | IAPV | NC_009025 | Dicistroviridae | ssRNA(+) | 9487 | 4665 | 6395 | N |
| Kashmir bee viurs | KBV | NC_004807 | Dicistroviridae | ssRNA(+) | 9506 | 4662 | 6407 | N |
| Lake Sinai virus 1 | LSV1 | KY465702 | Sinhaliviridae | ssRNA(+) | 5900 | 2220 | 3716 | Y |
| Lake Sinai virus 2 | LSV2 | MT732482 | Sinhaliviridae | ssRNA(+) | 5901 | 2221 | 3717 | N |
| Lake Sinai virus 3 Liaoning | LSV3 Liaoning | MZ821904 | Sinhaliviridae | ssRNA(+) | 6160 | 2150 | 3715 | Y |
| Lake Sinai virus 3 Shanxi | LSV3 Shanxi | MZ821851 | Sinhaliviridae | ssRNA(+) | 6024 | 2149 | 3714 | N |
| Lake Sinai virus 4 | LSV4 | MZ821893 | Sinhaliviridae | ssRNA(+) | 6036 | 2616 | 3413 | Y |
| Lake Sinai virus Liverpool | LSV Lpool | **PV239929** | Sinhaliviridae | ssRNA(+) | 5878 | 2117 | 3691 | Y |
| Lake Sinai virus 8 | LSV8 China | MZ821873 | Sinhaliviridae | ssRNA(+) | 6071 | 2138 | 3712 | N |
| Lake Sinai virus France | LSV France | **PV239928** | Sinhaliviridae | ssRNA(+) | 6790 | 2599 | 3396 | Y |
| Lake Sinai virus 3 Scilly | LSV3 Scilly | **PV239927** | Sinhaliviridae | ssRNA(+) | 6063 | 2138 | 3703 | Y |
| Lake Sinai virus strain | LSV | KM886903 | Sinhaliviridae | ssRNA(+) | 5187 | 2424 | 3221 | Y |
| Lake Sinai virus Tonga variant Belle-Ile | LSV TO | **PV239930** | Sinhaliviridae | ssRNA(+) | 5913 | 2193 | 3683 | Y |
| Leuven Picorna-like virus 3 | LPV3 | MZ443606 | Iflaviridae | ssRNA(+) | 10442 | 8497 | 10188 | N |
| Mayfield virus 1 | MfV1 | MH614304 | Picornavirales | ssRNA(+) | 8948 | 4688 | 5809 | Y |
| Mayfield virus 2 | MfV2 | **PV239931** | Picornavirales | ssRNA(+) | 8959 | 4703 | 5824 | Y |
| Mill Lade virus | MLV | MH614306 | uncl. Riboviria | NA | 3152 | 145 | 1668 | Y |
| Sacbrood virus | SBV | **PV239932** | Iflaviridae | ssRNA(+) | 8830 | 7005 | 8750 | Y |
| Sacbrood virus-like virus | SBV-like | **PV239933** | Iflaviridae | ssRNA(+) | 8803 | 6977 | 8722 | Y |
| Slow bee paralysis virus strain Harpenden | SBPV harp | GU938761 | Iflaviridae | ssRNA(+) | 9413 | 7575 | 9140 | Y |
| Slow bee paralysis virus strain Rothamsted | SBPV roth | EU035616 | Iflaviridae | ssRNA(+) | 9484 | 7643 | 9208 | Y |
| Vespa velutina associated acypi-like virus | VvAV | MN565042 | uncl. viruses | NA | 9900 | 5080 | 6237 | N |
| Victoria bee virus 2 | VBV2 | MG995724 | uncl. Picornavirales | ssRNA(+) | 8874 | 7123 | 8649 | N |

Table S4: Putative novel viruses found in this study. Viruses were considered novel when they shared less than 90% RdRp protein identity with previously described species. Sampling site and collection year of positive libraries (numbered according to table S1) are shown with normalized average fold coverage. The blastx search results show the closest match, identity and the query coverage, and the accession number of the closest match when using only the RdRp sequences with % amino acid identity. Iflavirus 1 and 2 have 81% amino acid identity and 91% nucleic acid identity. Virus acronyms: Lake Sinai virus (LSV), sacbrood virus (SBV).

| Name | Site and year | Fold coverage | Genome size (bp) | Blastx search | | | | GenBank accession |
| --- | --- | --- | --- | --- | --- | --- | --- | --- |
|  |  |  |  | Organism [accession] | Identity | Query cover | RdRp match (ident.) |  |
| Bt Iflavirus 1 | Belle-Ile 2015 (35)  2021 (14)  Quiberon 2015 (43)  2021 (21)  Liverpool 2015 (40)  Penryn 2015 (42) | 26,222  54  19,344  20,357  2  41 | 9,233 | Iflaviridae sp. [XCO48714] | 47 % | 94 % | AWK77843  (65 %) | PV239922 |
| Bt Iflavirus 2 | Alderney 2021 (13)  Belle-Ile 2015 (25)  2021 (14)  Guernsey 2021 (15)  Isle of Man 2021 (16)  Le Conquet 2015 (39)  2021 (17)  Liverpool 2021 (18)  Ouessant 2021 (19)  Penryn 2015 (42)  2021 (20)  Quiberon 2015 (32) | 79,501  853  7  15  99  3,398  35,394  12  2  7  6  3,513 | 9,229 | Iflaviridae sp. [XCO48714] | 47 % | 95 % | AWK77843  (65 %) | PV239923 |
| Bt Iflavirus 3 | Alderney (13)  Belle-Ile (14)  Guernsey (15)  Isle of Man (16)  Le Conquet (17)  Liverpool (18)  Ouessant (19)  Penryn (20)  all 2021 | 6  5  16  125  43,185  4  1  4 | 8,927 | MG995699/ Darwin bee virus 4 [AWK77851] | 59 % | 95 % | AWK77851 (65 %) | PV239924 |
| SBV-like virus | all *A. mellifera*  fold coverage >100  Alderney 2015 (23)  Alderney 2021 (2)  Belle-Ile 2015 (24)  Belle-Ile 2021 (3)  Le Conquet (7)  6 *B. terrestris* | avr 9,287  1,125  178  119,9478  61,760  30,985  avr 19 | 8,803 | Sacbrood virus (South Korea) [OR496425] | 76 % | 97 % | QKW94194 (88 %) | PV239933 |
| LSV Liverpool | Liverpool 2021 (8) | 140 | 5,878 | Lake Sinai virus 8 [OM744325] | 78 % | 99 % | UDY81098  (89 %) | PV239929 |
| LSV France | Alderney 2015 (23)  2021 (2)  Belle-Ile 2015 (24)  Le Conquet 2015 (28)  Quiberon 2021 (11)  Cherbourg 2015 (25) | 33  49  10  39  203  148 | 6,790 | Lake Sinai virus 3 [OP972891] | 77 % | 99 % | QSH48595  (88 %) | PV239928 |

Table S5: *De novo* assembled contigs used as reference genomes for virus mapping. Sampling site and collection year of positive libraries (numbered according to table S1) are shown with normalized average fold coverage. The blast search results show the closest match, identity and the query coverage. Virus acronyms: Black queen cell virus (BQCV), deformed wing virus (DWV), Mayfield virus 2 (MfV2), Lake Sinai virus (LSV), sacbrood virus (SBV). Note that except for MfV2 *Apis mellifera* libraries showed much higher average fold coverage than *Bombus terrestris*.

| Name | Libraries | Fold coverage | Genome size (bp) | Blastn search | | | GenBank accession |
| --- | --- | --- | --- | --- | --- | --- | --- |
|  |  |  |  | Organism [accession] | Identity | Query cover |  |
| DWV-B | all *A. mellifera*  3 *B. terrestris* | 177,634  39 | 10,127 | DWV-B [OR361560] | 99.85 % | 100 % | PV239926 |
| DWV-A | all *A. mellifera*  3 *B. terrestris* | 67,395  7 | 10,141 | DWV-A [OR361533] | 99 % | 100 % | PV239925 |
| BQCV Cherbourg | 11 *A. mellifera*  Le Conquet 2021 *B. ter.* | 5,233  52 | 8,429 | BQCV [EF517519] | 93 % | 99 % | PV239921 |
| BQCV Belle-Ile | 12 *A. mellifera*  Liverpool 2015 + 2021 *B. ter.* | 30,777  60 | 8,436 | BQCV [MT416539] | 97 % | 100 % | PV239920 |
| LSV3 Scilly | Scilly Isles 2021 (12)  Guernsey 2015 (26)  Liverpool 2015 (29)  Penryn 2015 (21)  Quiberon 2015 (32),  all *A. mellifera* | 1,070  202  23  754  252 | 6,063 | LSV3 [MZ821904] | 87 % | 100 % | PV239927 |
| LSV-TO Belle-Ile | Belle-Ile *A. mel* 2015 (24) | 211 | 5,913 | LSV TO [NC_035116] | 81 % | 99 % | PV239930 |
| MfV2 | Guernsey 2015 *B.ter.* | 6,361 | 8,959 | MfV2 [MH614305] | 94 % | 98 % | PV239931 |
| SBV | 13 *A. mellifera*  4 *B. terrestris* | 9,575  6 | 8,830 | SBV [MT636329] | 98 % | 100 % | PV239932 |

Table S6: RdRp similarity within the Dicistroviridae used in our reference database. Viruses include: acute bee paralysis virus (ABPV, not detected), Aphis gossypii virus (AGV), Aphid lethal paralysis virus (ALPV, not detected), black queen cell virus (BQCV), Israeli acute paralysis virus (IAPV, not detected) and Kashmir bee virus (KBV, not detected). RdRp sequences with >80% identity are highlighted in blue

|  | BQCV BI | ALPV | AGV | KBV | ABPV | BQCV Cherb. | IAPV |
| --- | --- | --- | --- | --- | --- | --- | --- |
| BQCV BI | - |  |  |  |  |  |  |
| ALPV | 37% | - |  |  |  |  |  |
| AGV | 39% | 56% | - |  |  |  |  |
| KBV | 39% | 43% | 42% | - |  |  |  |
| ABPV | 40% | 44% | 41% | 73% | - |  |  |
| BQCV Cherb. | 89% | 36% | 38% | 39% | 39% | - |  |
| IAPV | 39% | 42% | 41% | 86% | 75% | 39% | - |

Table S7: RdRp similarity within the Iflaviridae used in our reference database. Viruses include: Bombus terrestris iflavirus (Bt Ifl.) 1,2 and 3, deformed wing virus (DWV) type A, B, C and D, sacbrood virus (SBV), SBV-like virus, slow bee paralysis virus (SBPV) Harpenden and Rothamsted. All Iflaviridae included in this table were also detected in the study. RdRp sequences with >80% identity are highlighted in blue.

|  | Bt Ifl. 2 | SBV | LPV3 | DWV-B | SBPV roth | Bt Ifl. 1 | DWV-A | DWV-C | DWV-D | Bt Ifl. 3 | SBV-like  virus | SBPV harp |
| --- | --- | --- | --- | --- | --- | --- | --- | --- | --- | --- | --- | --- |
| Bt Iflavirus 2 | - |  |  |  |  |  |  |  |  |  |  |  |
| SBV | 63% | - |  |  |  |  |  |  |  |  |  |  |
| LPV3 | 40% | 41% | - |  |  |  |  |  |  |  |  |  |
| DWV-B | 40% | 42% | 48% | - |  |  |  |  |  |  |  |  |
| SBPV roth | 37% | 38% | 56% | 44% | - |  |  |  |  |  |  |  |
| Bt Iflavirus 1 | 82% | 62% | 39% | 40% | 36% | - |  |  |  |  |  |  |
| DWV-A | 40% | 41% | 47% | 85% | 45% | 40% | - |  |  |  |  |  |
| DWV-C | 40% | 42% | 47% | 80% | 44% | 39% | 80% | - |  |  |  |  |
| DWV-D | 40% | 42% | 47% | 82% | 44% | 40% | 81% | 89% | - |  |  |  |
| Bt Iflavirus 3 | 67% | 64% | 40% | 40% | 38% | 67% | 40% | 40% | 41% | - |  |  |
| SBV-like virus | 63% | 77% | 42% | 40% | 37% | 64% | 40% | 40% | 41% | 64% | - |  |
| SBPV harp | 36% | 38% | 56% | 44% | 83% | 37% | 44% | 45% | 45% | 37% | 38% | - |

Table S8: RdRp similarity within the Rhabdoviridae used in our reference database. Viurses include: Apis rhabdovirus 1, 2 (not detected) and 5 (not detected).

|  | ARV5 | ARV1 | ARV2 |
| --- | --- | --- | --- |
| ARV5 | - |  |  |
| ARV1 | 43% | - |  |
| ARV2 | 34% | 42% | - |

Table S9: RdRp similarity within the Sinhaliviridae used in our reference database. All viruses included are Lake Sinai viruses (LSV). LSV2, LSV3 Shanxi and LSV8 China were not detected. RdRp sequences with >80% identity are highlighted in blue.

|  | LSV Lpool | LSV1 | LSV France | LSV2 | LSV | LSV3 Scilly | LSV TO | LSV3 Shanxi | LSV3 Liaoning | LSV4 | LSV8 China |
| --- | --- | --- | --- | --- | --- | --- | --- | --- | --- | --- | --- |
| LSV Lpool | - |  |  |  |  |  |  |  |  |  |  |
| LSV1 | 67% | - |  |  |  |  |  |  |  |  |  |
| LSV France | 69% | 75% | - |  |  |  |  |  |  |  |  |
| LSV2 | 69% | 72% | 71% | - |  |  |  |  |  |  |  |
| LSV | 52% | 54% | 54% | 54% | - |  |  |  |  |  |  |
| LSV3 Scilly | 68% | 76% | 74% | 72% | 55% | - |  |  |  |  |  |
| LSV TO | 68% | 71% | 70% | 78% | 53% | 70% | - |  |  |  |  |
| LSV3 Shanxi | 69% | 75% | 76% | 72% | 54% | 85% | 71% | - |  |  |  |
| LSV3 Liaoning | 69% | 75% | 75% | 72% | 55% | 88% | 71% | 83% | - |  |  |
| LSV4 | 51% | 55% | 56% | 54% | 89% | 55% | 52% | 55% | 55% | - |  |
| LSV8 China | 75% | 69% | 67% | 67% | 51% | 68% | 69% | 70% | 69% | 52% | - |

Table S10: RdRp similarity for viruses with unknon families used in our reference database. Viruses include: uir hill virus (AMV) 1, 2 (not detected) and 3, Boghill burn virus (BBV, not detected), Dumyat virus (DV), Gorebridge virus (GV, not detected), Mayfield virus (MfV) 1 and 2, Mill Lade virus (MLV), Vespa velutina associated acypii-like virus (VvAV, not detected) and Victoria bee virus 2 (VBV2, not detected). RdRp sequences with >80% identity are highlighted in blue.

|  | AMV1 | AMV2 | AMV3 | BBV | DV | GV | MfV1 | MfV2 | MLV | VvAV | VBV2 |
| --- | --- | --- | --- | --- | --- | --- | --- | --- | --- | --- | --- |
| AMV1 | - |  |  |  |  |  |  |  |  |  |  |
| AMV2 | 80% | - |  |  |  |  |  |  |  |  |  |
| AMV3 | 79% | 84% | - |  |  |  |  |  |  |  |  |
| BBV | 23% | 22% | 22% | - |  |  |  |  |  |  |  |
| DV | 19% | 19% | 19% | 15% | - |  |  |  |  |  |  |
| GV | 27% | 25% | 26% | 24% | 21% | - |  |  |  |  |  |
| MfV1 | 19% | 18% | 19% | 22% | 18% | 32% | - |  |  |  |  |
| MfV2 | 19% | 19% | 19% | 21% | 17% | 30% | 85% | - |  |  |  |
| MLV | 49% | 48% | 46% | 25% | 18% | 30% | 21% | 21% | - |  |  |
| VvAV | 24% | 23% | 22% | 89% | 16% | 26% | 21% | 21% | 25% | - |  |
| VBV2 | 27% | 25% | 25% | 22% | 25% | 39% | 27% | 27% | 29% | 22% | - |

Table S11: Primers used to detect breakpoints in the deformed wing virus (DWV) genome. The genome position shows the primer binding site relative to the DWV-B genome (PV239926). Each primer pair (F and R) targets different DWV variants. Recombinant amplicons covering breakpoints 1 to 3 were confirmed using Sanger sequencing while 4 and 5 showed no recombinant sequences.

| Breakpoint | Genome positon (bp) | DWV variant | Sequence 5’ to 3’ |
| --- | --- | --- | --- |
| 1 (B 🡪 A) | 375 | DWV-B | F: GAATGCCATGTGACCGCTCA |
|  | 1,727 | DWV-A | R: TGACGTCTCAACATCCTAACCTG |
| 2 (A 🡪 B) | 1,445 | DWV-A | F: GGCCTATCAAAGAGCATGTCCC |
|  | 2,789 | DWV-B | R: CAGTCATATCTTCATCAGGCGCA |
| 3 (B 🡪 A) | 4,992 | DWV-B | F: TATCACTTGGCGACGCAACC |
|  | 5,618 | DWV-A | R: TCCAGATGCACCACACATGC |
| 4 (A 🡪 B) | 6,163 | DWV-A | F: ATGGCTAACCGTCGTAAGGC |
|  | 6,501 | DWV-B | R: TGCGTTCATTCCTGCTGCAT |
| 5 (B 🡪 A) | 7,686 | DWV-B | F: TGAGTCGACGGCTGCTTTTC |
|  | 7,830 | DWV-A | R: TCCCGCGAGACCACCATAAT |

Table S12: Deformed wing virus type A and B (DWV-A and DWV-B) detection in individual *Apis mellifera* and *Bombus terrestris* using RT-PCR compared with pools used in RNA-seq. The PCR columns show the number of individuals that tested positive for either DWV-A or DWV-B, and the co-infection shows the number of bees that were positive for both variants. The % genome columns show the reads covering the DWV genome (and RNA dependen RNA polymerase [RdRp]) in libraries from sample pools, and fold coverage shows the average read depth across the genome. Samples for which PCR indicated DWV presence but coverage did not meet criteria for ‘detection’ in RNA-seq data are presented in grey font. Covergae below 10% (RdRp) are not shown.

| Year | Species | Site | N individuals | Virus | PCR | co-infection | % genome (% RdRp) | Fold coverage |
| --- | --- | --- | --- | --- | --- | --- | --- | --- |
| 2021 | *A. mel* | Arran | 30 | DWV-A | 6 | 6 | 64% (100%) | 474928 |
|  |  |  |  | DWV-B | 29 |  | 97% (100%) | 412489 |
|  |  | Alderney | 28 | DWV-A | 0 | 0 | 57% (100%) | 373 |
|  |  |  |  | DWV-B | 3 |  | 92% (100%) | 462 |
|  |  | Belle-Ile | 30 | DWV-A | 2 | 2 | 72% (100%) | 107988 |
|  |  |  |  | DWV-B | 11 |  | 100% (100%) | 128969 |
|  |  | Cherbourg | 30 | DWV-A | 2 | 2 | 75% (100%) | 51445 |
|  |  |  |  | DWV-B | 30 |  | 100% (100%) | 13848 |
|  |  | Guernsey | 30 | DWV-A | 15 | 15 | 100% (100%) | 413523 |
|  |  |  |  | DWV-B | 30 |  | 100% (100%) | 666647 |
|  |  | Isle of Man | 30 | DWV-A | 0 | 0 | 58% (100%) | 181 |
|  |  |  |  | DWV-B | 0 |  | 94% (100%) | 371 |
|  |  | Le Conquet | 30 | DWV-A | 6 | 6 | 77% (100%) | 110348 |
|  |  |  |  | DWV-B | 30 |  | 100% (100%) | 328145 |
|  |  | Liverpool | 30 | DWV-A | 3 | 3 | 66% (100%) | 58541 |
|  |  |  |  | DWV-B | 26 |  | 100% (100%) | 45076 |
|  |  | Ouessant | 30 | DWV-A | 3 | 3 | 67% (100%) | 141155 |
|  |  |  |  | DWV-B | 29 |  | 100% (100%) | 371946 |
|  |  | Penryn | 30 | DWV-A | 7 | 7 | 71% (100%) | 77238 |
|  |  |  |  | DWV-B | 30 |  | 100% (100%) | 158136 |
|  |  | Quiberon | 30 | DWV-A | 10 | 8 | 63% (100%) | 94 |
|  |  |  |  | DWV-B | 27 |  | 94% (100%) | 123948 |
|  |  | Scilly Isles | 30 | DWV-A | 0 | 0 | 59% (100%) | 25 |
|  |  |  |  | DWV-B | 1 |  | 85% (100%) | 38 |
| 2021 | *B. ter* | Alderney | 30 | DWV-A | 1 | 0 |  |  |
|  |  |  |  | DWV-B | 0 |  |  |  |
|  |  | Belle-Ile | 30 | DWV-A | 1 | 0 |  |  |
|  |  |  |  | DWV-B | 6 |  | 6% (19%) |  |
|  |  | Guernsey | 17 | DWV-A | 0 | 0 | 16% (46%) |  |
|  |  |  |  | DWV-B | 14 |  | 24% (73%) |  |
|  |  | Isle of Man | 30 | DWV-A | 0 | 0 | - |  |
|  |  |  |  | DWV-B | 0 |  | - |  |
|  |  | Le Conquet | 30 | DWV-A | 0 | 0 | - |  |
|  |  |  |  | DWV-B | 12 |  | 4% (12%) |  |
|  |  | Liverpool | 30 | DWV-A | 0 | 0 | 20% (53%) |  |
|  |  |  |  | DWV-B | 17 |  |  |  |
|  |  | Ouessant | 30 | DWV-A | 0 | 0 | 4% (14%) |  |
|  |  |  |  | DWV-B | 10 |  | 11% (43%) |  |
|  |  | Penryn | 30 | DWV-A | 0 | 0 | 4% (15%) |  |
|  |  |  |  | DWV-B | 9 |  | 6% (12%) |  |
|  |  | Quiberon | 30 | DWV-A | 0 | 0 | 57% (100%) | 14 |
|  |  |  |  | DWV-B | 13 |  | 81% (100%) | 23 |
|  |  | Scilly Isles | 30 | DWV-A | 0 | 0 | 64% (90%) | 5 |
|  |  |  |  | DWV-B | 0 |  | 60% (100%) | 73 |
|  | | | | | | | | |
| Table S12 continued | |  |  |  |  |  |  |  |
| 2015 | *A. mel* | Alderney | 30 | DWV-A | 0 | 0 | 70% (100%) | 306 |
|  |  |  |  | DWV-B | 0 |  | 96% (100%) | 5271 |
|  |  | Belle-Ile | 28 | DWV-A | 19 | 19 | 87% (100%) | 30321 |
|  |  |  |  | DWV-B | 27 |  | 100% (100%) | 563324 |
|  |  | Cherbourg | 30 | DWV-A | 15 | 14 | 100% (100%) | 83455 |
|  |  |  |  | DWV-B | 27 |  | 100% (100%) | 451506 |
|  |  | Guernsey | 22 | DWV-A | 1 | 1 | 50% (99%) | 14 |
|  |  |  |  | DWV-B | 4 |  | 100% (100%) | 64769 |
|  |  | Isle of Man | 30 | DWV-A | 0 | 0 | 39% (90%) | 2 |
|  |  |  |  | DWV-B | 6 |  | 75% (100%) | 247 |
|  |  | Le Conquet | 30 | DWV-A | 3 | 3 | 37% (93%) | 4 |
|  |  |  |  | DWV-B | 22 |  | 100% (100%) | 130018 |
|  |  | Liverpool | 29 | DWV-A | 4 | 3 | 57% (100%) | 108 |
|  |  |  |  | DWV-B | 12 |  | 100% (100%) | 25665 |
|  |  | Ouessant | 30 | DWV-A | 0 | 0 | 43% (98%) | 5 |
|  |  |  |  | DWV-B | 5 |  | 68% (100%) | 72 |
|  |  | Penryn | 30 | DWV-A | 6 | 6 | 51% (99%) | 3 |
|  |  |  |  | DWV-B | 10 |  | 100% (100%) | 241677 |
|  |  | Quiberon | 30 | DWV-A | 7 | 7 | 45% (97%) | 32 |
|  |  |  |  | DWV-B | 26 |  | 100% (100%) | 352930 |
|  |  | Scilly Isles | 30 | DWV-A | 0 | 0 | 38% (96%) | 1 |
|  |  |  |  | DWV-B | 0 |  | 63% (100%) | 33 |
| 2015 | *B. ter* | Alderney | 30 | DWV-A | 0 | 0 | 38% (81%) | 2 |
|  |  |  |  | DWV-B | 0 |  | 64% (100%) | 22 |
|  |  | Belle-Ile | 30 | DWV-A | 0 | 0 | - |  |
|  |  |  |  | DWV-B | 14 |  | 8% (40%) |  |
|  |  | Cherbourg | 30 | DWV-A | 2 | 0 |  |  |
|  |  |  |  | DWV-B | 6 |  |  |  |
|  |  | Guernsey | 30 | DWV-A | 0 | 0 |  |  |
|  |  |  |  | DWV-B | 1 |  |  |  |
|  |  | Isle of Man | 30 | DWV-A | 1 | 0 |  |  |
|  |  |  |  | DWV-B | 3 |  |  |  |
|  |  | Le Conquet | 30 | DWV-A | 0 | 0 |  |  |
|  |  |  |  | DWV-B | 6 |  |  |  |
|  |  | Liverpool | 30 | DWV-A | 1 | 0 |  |  |
|  |  |  |  | DWV-B | 1 |  |  |  |
|  |  | Ouessant | 13 | DWV-A | 0 | 0 |  |  |
|  |  |  |  | DWV-B | 2 |  |  |  |
|  |  | Penryn | 30 | DWV-A | 0 | 0 |  |  |
|  |  |  |  | DWV-B | 0 |  |  |  |
|  |  | Quiberon | 30 | DWV-A | 0 | 0 |  |  |
|  |  |  |  | DWV-B | 0 |  |  |  |
|  |  | Scilly Isles | 30 | DWV-A | 0 | 0 |  |  |
|  |  |  |  | DWV-B | 0 |  |  |  |

## Figures


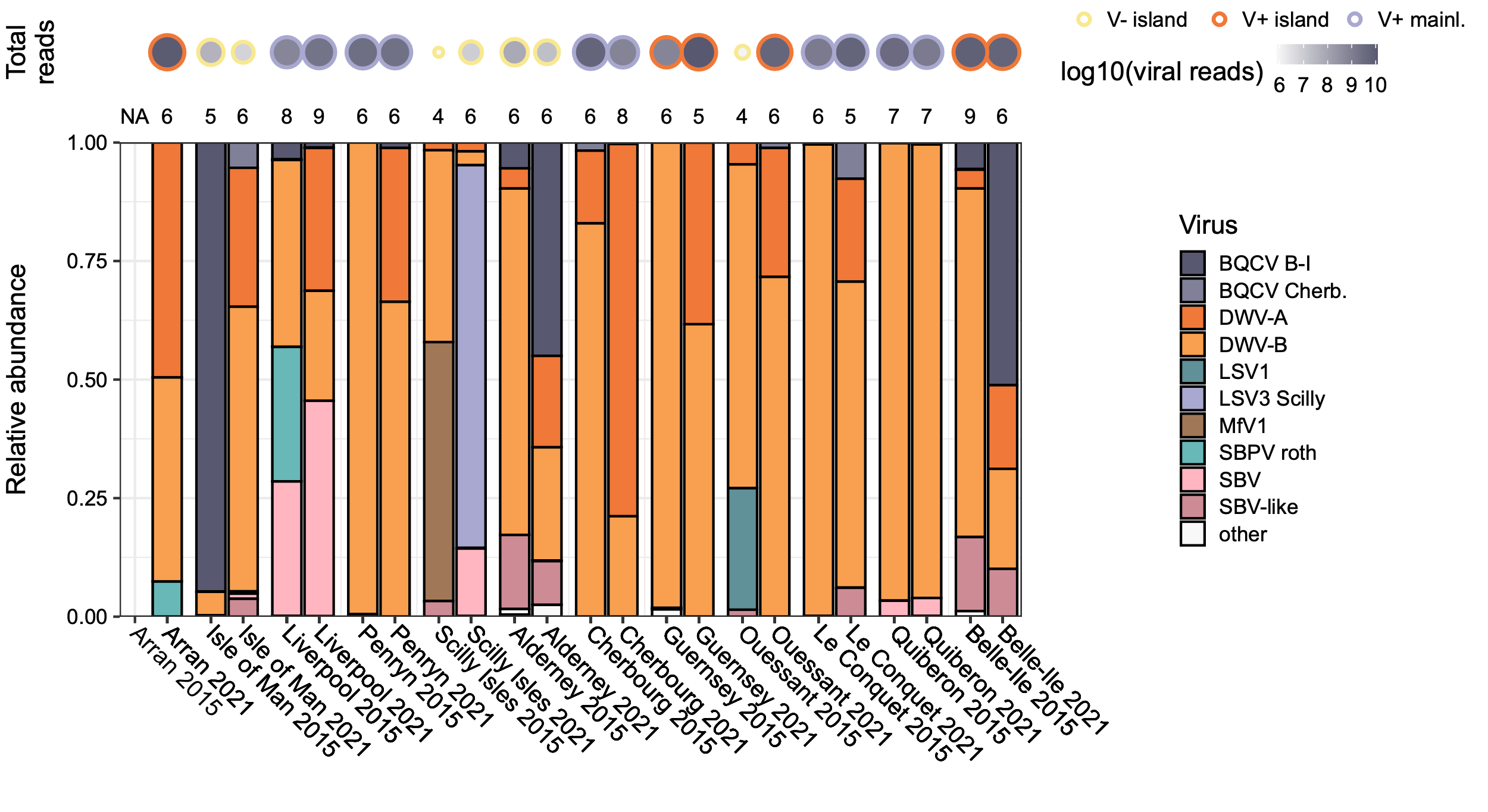


Figure S1: Most abundant viruses in *Apis mellifera* viromes from 12 sites from two years. Coloured bars show the 10 most abundant viruses, including black queen cell virus (BQCV) Belle-Ile and Cherbourg variant, deformed wing virus type A and B (DWV-A and DWV-B), Lake Sinai virus 1 and 3 Scilly variant (LSV1 and LSV3 Scilly), Mayfield virus 1 (MfV1), slow bee paralysis virus strain Rothamsted (SBPV roth), sacbrood virus (SBV) and SBV-like virus. Other viruses were: Aphis gossypii virus, Apis rhabdo-like viruses 1, bee Macula-like virus, LSV, LSV France, LSV Liverpool, LSV3 Liaoning, LSV4, LSV Tonga and SBPV Harpenden. The sites are sorted north to south, with the 2015 sample on the left and the 2021 sample on the right. The upper panel shows the number of viruses found in each library, and the circles indicate the number of viral reads (log10 transformed) and are outlined by a colour indicating whether the site is a varroa-free (V-) or varroa-present (V+) island or the V+ mainland. Note that Arran was only sampled in 2021.


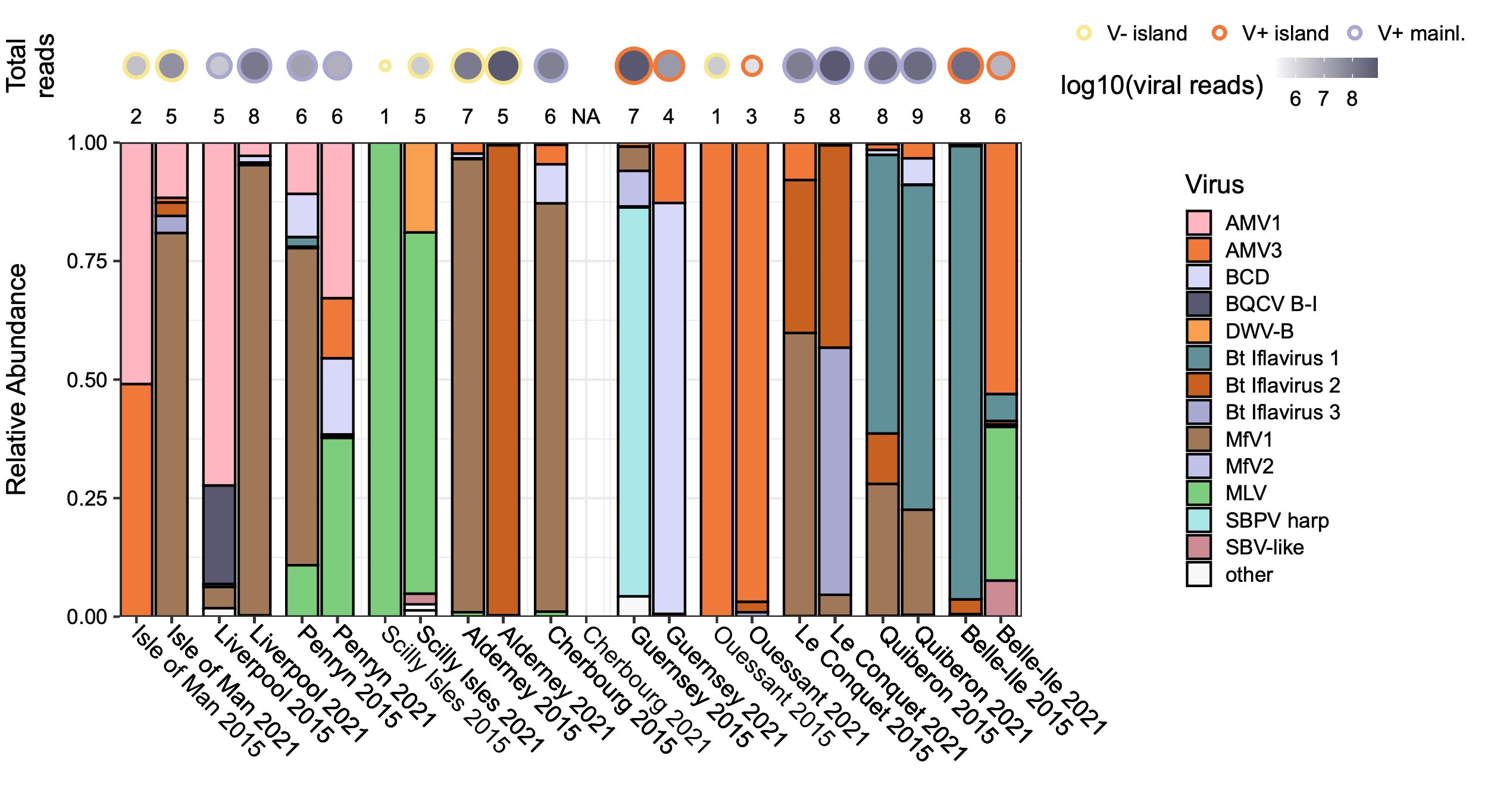


Figure S2: Most abundant viruses in *Bombus terrestris* viromes from 11 sites from two years. Coloured bars show the 14 most abundant viruses, including Allemuir hill virus 1 and 3 (AMV1, AMV3), Bombus cryptarum desnovirus (BCD), black queen cell virus Belle-Ile variant (BQCV B-I), deformed wing virus type B (DWV-B), three novel Bombus terrestris Iflaviridae (Bt Iflavirus 1 to 3), Mayfield virus 1 and 2 (MfV1, MfV2), Mill lade virus (MLV), slow bee paralysis virus Harpenden (SBPV harp), sacbrood virus-like virus (SBV-like). Other viruses were: Aphis gossypii virus, BQCV Cherbourg variant, DWV-A, SBV and SBPV Rothamsted. The sites are sorted from north to south with the 2015 sample on the left and 2021 on the right. The upper panel shows the number of viruses found in each library and the circles indicate the number of viral reads (log10 transformed) and are outlined by a colour indicating whether the site is a varroa-free (V-) or varroa-present (V+) island or the V+ mainland. Note that *B. terrestris* was not collected from Cherbourg in 2021.


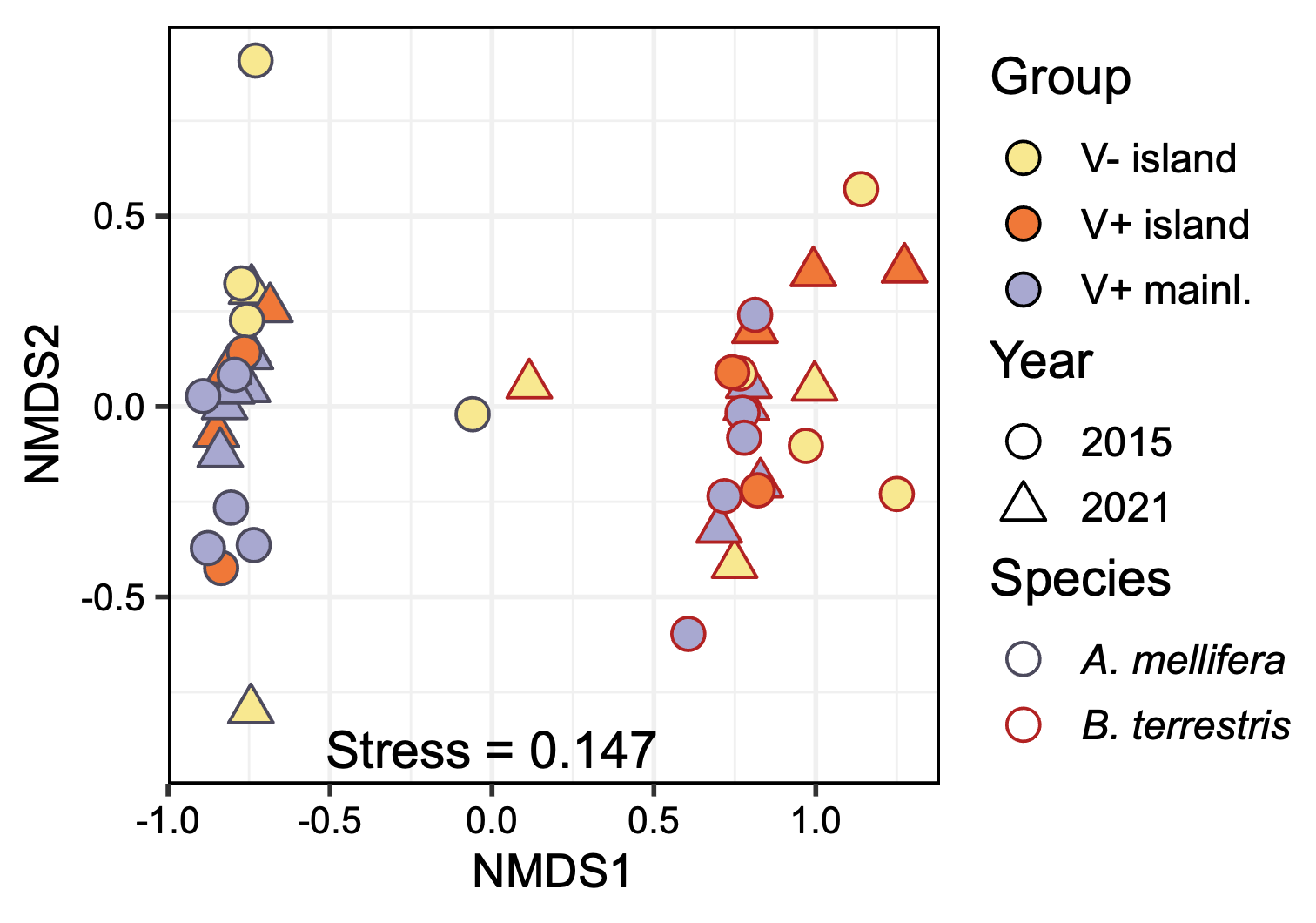


Figure S3: Non-metric Multi-Dimensional Scaling (NMDS) ordination plots, based on Bray-Curtis distance matrices of *Apis mellifera* and *Bombus terrestris* viromes. Shapes indicate the sampling year (triangles 2015 or circles 2021) and colours whether bees were collected on a varroa-free (yellow) or varroa-present (orange) islands (V- or V+ island) or the varroa-present mainland (V+ mainl., purple). The outline colour of the shape shows the two different host species (*A. mellifera*: black or *B. terrestris*: red).


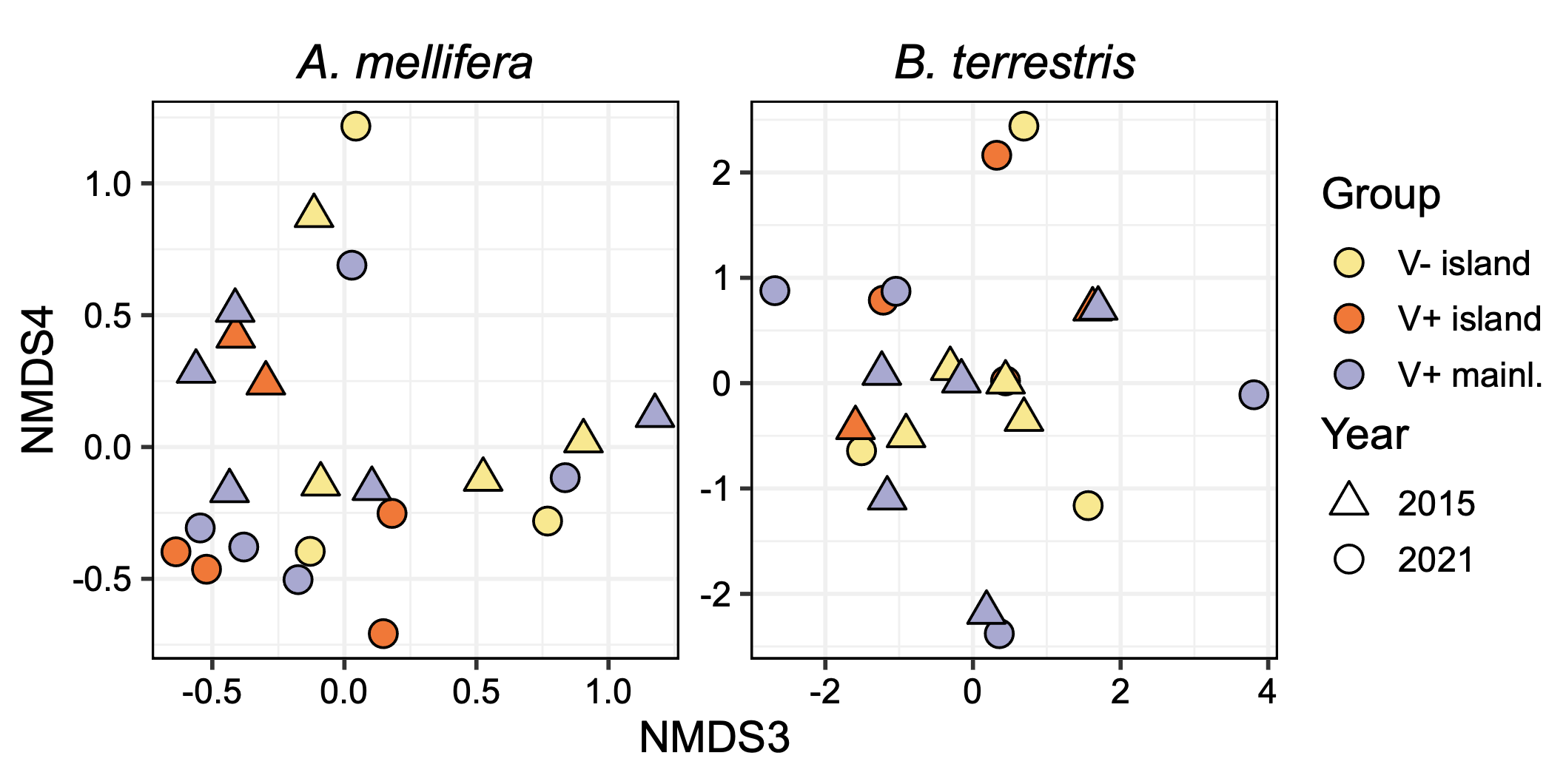


Figure S4: Non-metric Multi Dimensional Scaling (NMDS) ordination plots, based on Bray-Curtis distance matrices of *Apis mellifera* and *Bombus terrestris* viromes. Showing NMDS3 and NMDS4. Stress in *A. mellifera* MDS = 0.057 and in *B. terrestris* MDS = 0.168. Shapes indicate the sampling year (triangles 2015 or circles 2021) and colours whether bees were collected on a varroa-free (yellow) or varroa-present (orange) island (V- or V+ island) or on the varroa-present mainland (V+ mainl., purple).


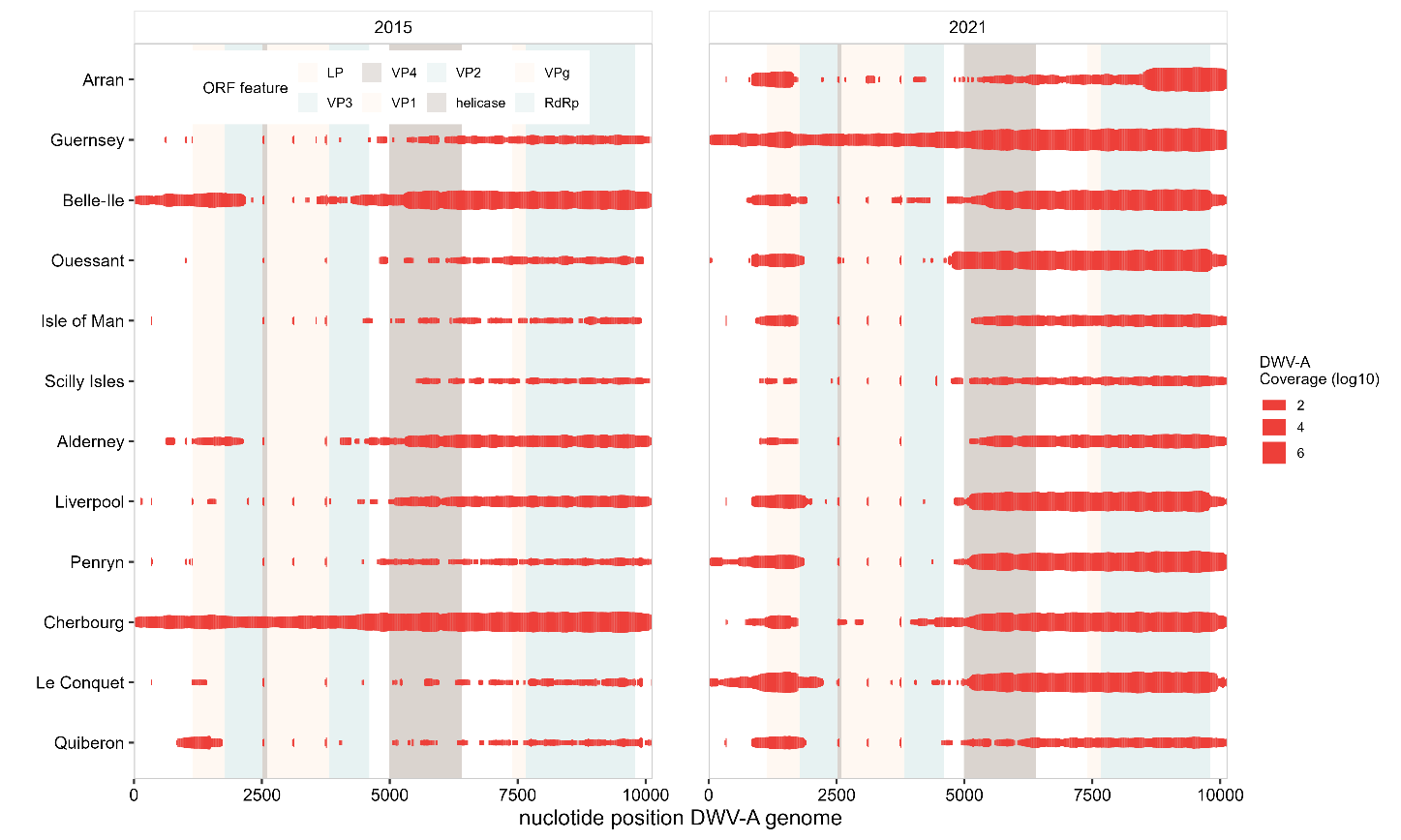


Figure S5: Deformed wing virus A (DWV-A) read coverage and variant in *Apis mellifera* viral communities in 2015 and 2021.. The line width indicates the log10-transformed coverage. The left upper panel shows a map of the DWV genome: 5’UTR, *leader protein* (*LP*) gene, *viral capsid protein* genes *VP2*, *VP4*, *VP1*, *VP3*, and the non-structural protein genes with the *helicase*, *VPg*, and *RNA-dependent RNA-polymerase* (*RdRp*) genes.


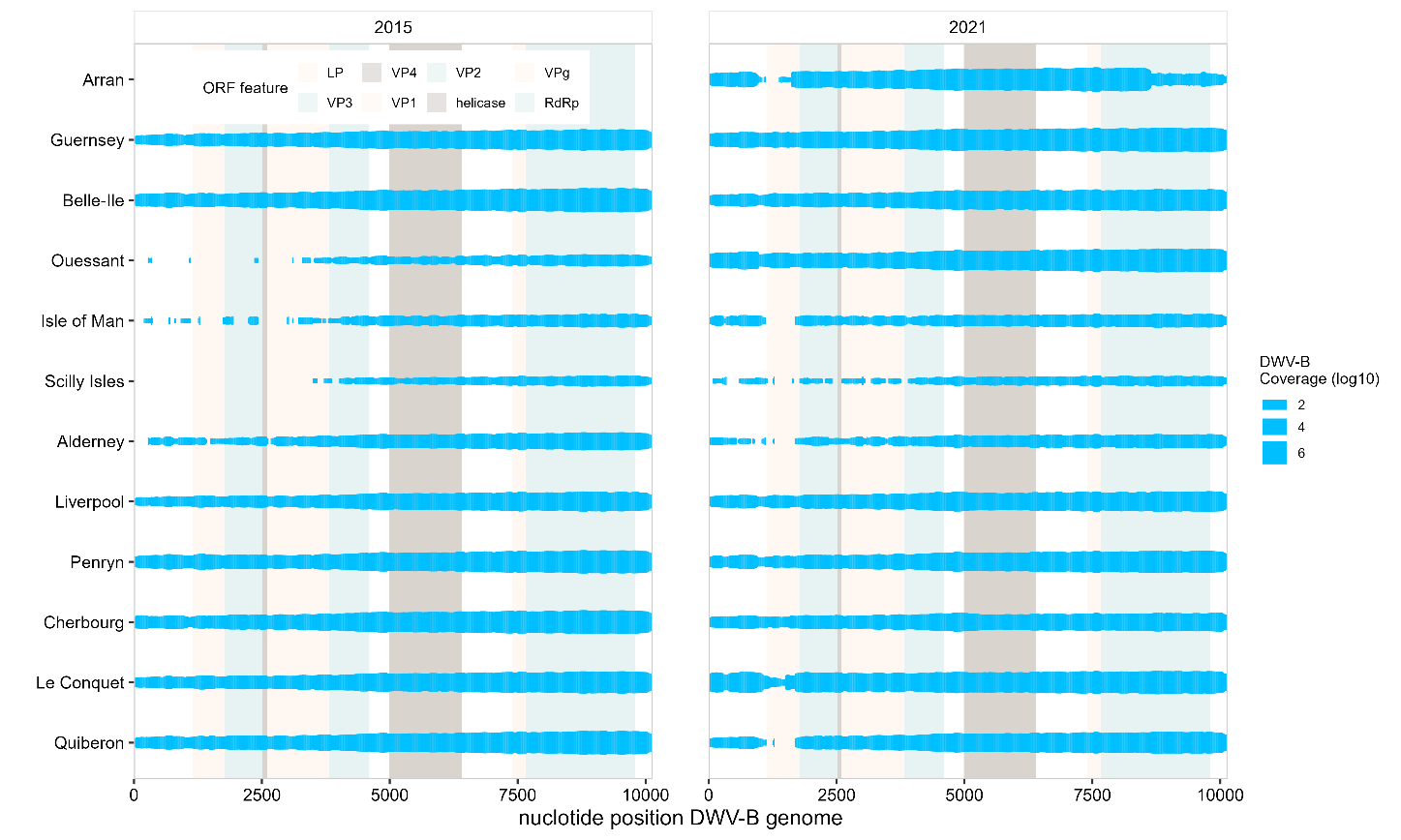


Figure S6: Deformed wing virus B (DWV-B) read coverage and variant in *Apis mellifera* viral communities in 2015 and 2021.. The line width indicates the log10-transformed coverage. The left upper panel shows a map of the DWV genome: 5’UTR, *leader protein* (*LP*) gene, *viral capsid protein* genes *VP2*, *VP4*, *VP1*, *VP3*, and the non-structural protein genes with the *helicase*, *VPg*, and *RNA-dependent RNA-polymerase* (*RdRp*) genes.

##
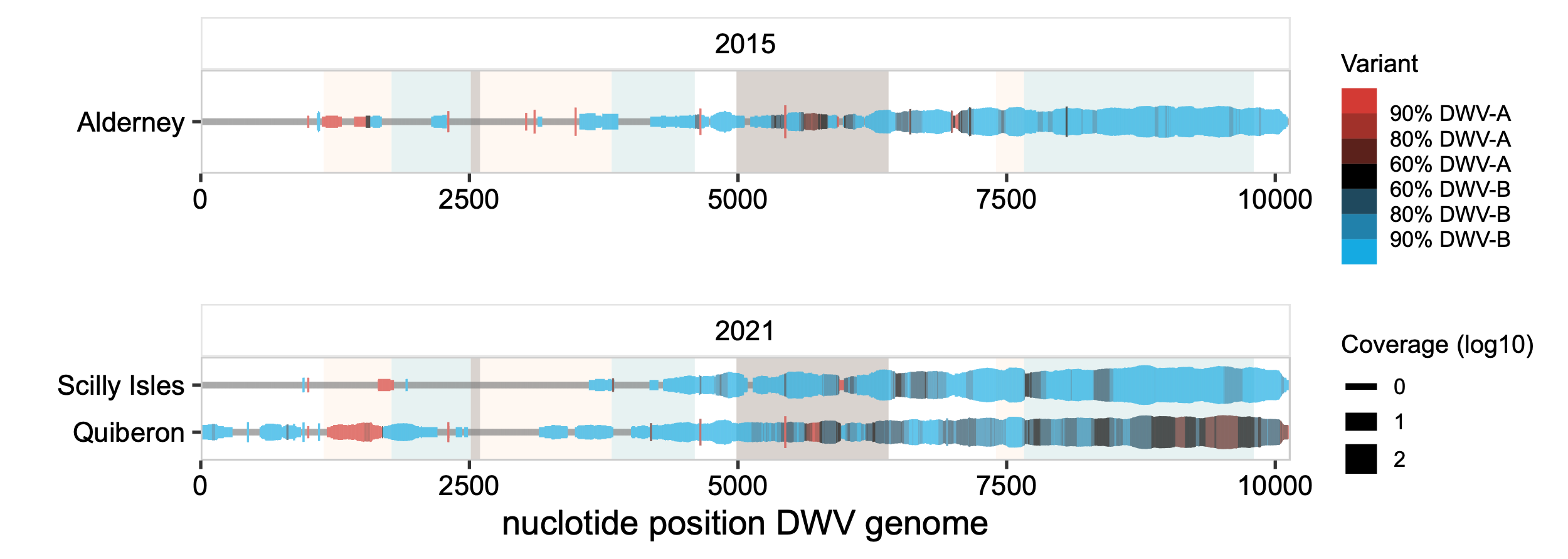


Figure S7: Deformed wing virus (DWV) read coverage and variant in *Bombus terrestris* viral communities in 2015 and 2021. The colours of the lines show the ratio of DWV-A to DWV-B reads at each nucleotide position in the DWV genome, with red colours indicating a majority of DWV-A reads, blue for a majority of DWV-B reads, and black for similar amounts of both variants. The line width indicates the log10-transformed coverage. The background colour indicates features of the DWV genome, from left to right: 5’UTR, *leader protein* (*LP*) gene (yellow), *viral capsid protein* genes *VP2* (blue), *VP4* (grey), *VP1* (yellow), *VP3* (blue), and the non-structural protein genes with the *helicase* (grey), *VPg* (yellow), and *RNA-dependent RNA-polymerase* (*RdRp*) (blue) genes.

## Data

S2: Sanger sequencing to verify recombinant sequences and determine breakpoint positions.

Alignments show the consensus sequences from three technical replicates aligned to the deformed wing virus (DWV) type B genome [PV239926]. Amplicons span regions containing breakpoints (highlighted in grey) in *Apis mellifera* samples from 2021. DWV-B is coloured in blue and DWV-A [PV239925] in red, letters show disagreements to the reference and dots indicate no differences to the reference.

Breakpoint 1

410 420 430 440 450 460 470 480 490 500

DWV-B TATGGTATAT CATTCGAAGT CGAATACTTG TGTATAGTTA TTGTATTTTA TTAGTAATAT TAGTAGTCCG TAACTATCAT AATCCTATTA TAGTTTGATT 496

DWV-A ......T... ..A....... T....GTA.T .A..AG.A.. ..A..C..A. .......... .......... .......T.. C.....T..T C........G 497

B1 Penryn ---------- ---------- ------.... .......... .......... .......... .......... .......... .......... ..........

B1 Liverpool ---------- ---------- ------.... .......... .......... .......... .......... .......... .......... ..........

B1 Quiberon ---------- ---------- -------... .......... .......... .......... .......... .......... .......... ..........

B1 Arran ---------- ---------- -------... .......... .......... .......... .......... .......... .......... ..........

B1 Guernsey ---------- ---------- ------.... .......... .......... .......... .......... .......... .......... ..........

B1 Le Conquet ---------- ---------- -------... .......... .......... .......... .......... .......... .......... ..........

B1 Ouessant ---------- ---------- -------... .......... .......... .......... .......... .......... .......... ..........

B1 Cherbourg ---------- ---------- ------.... .......... .......... .......... .......... .......... .......... ..........

510 520 530 540 550 560 570 580 590 600

DWV-B ATATGATAGA CCACTGCAGT ATCGAGTAGA GTTTAGAAAG AGTAGTGCAA TAGTAAGATC ACTGTCACCG ACCACTCATT GTAATAGTGA GGTTTGTCGG 596

DWV-A TG..A..... .......... .......... ....C...T. C......... .....TA... .......... ....TCT... .....GA.-. .A.C...... 596

B1 Penryn .......... .......... .......... .......... .......... .......... .......... .......... .......... ..........

B1 Liverpool .......... .......... .......... .......... .......... .......... .......... .......... .......... ..........

B1 Quiberon .......... .......... .......... .......... .......... .......... .......... .......... .......... ..........

B1 Arran .C........ .......... .......... .......... .......... .......... .......... .......... .......... ..........

B1 Guernsey .......... .......... .......... .......... .......... .......... .......... .......... .......... ..........

B1 Le Conquet .......... .......... .......... .......... .......... .......... .......... .......... .......... ..........

B1 Ouessant .......... .......... .......... .......... .......... .......... .......... .......... .......... ..........

B1 Cherbourg .......... .......... .......... .......... .......... .......... .......... .......... .......... ..........

610 620 630 640 650 660 670 680 690 700

DWV-B AAACCAGTTA TTGTGCAGCG ACTAGCAATC GTGAATCAAT ATAGTTGGTA TTCTAAATAT GAGACGATTC GGCGATTTTA TTGCGACTGA AATTTCATAT 696

DWV-A ......T.AT ..A..A..T. .......... A..G..T..A T...A..... .....GT.TA ...GT..... ....C.GCGG .-........ ..C...TA.A 695

B1 Penryn .......... .......... ..C....... .......... .......... .......... .......... .......... .......... ..........

B1 Liverpool .......... .......... .......... .......... .......... .......... .......... .......... ......T... ..........

B1 Quiberon .......... .......... .......... .......... .......... .......... .......... .......... .......... ..........

B1 Arran .......... .......... .......... .......... .......... .......... .......... .......... .......... ..........

B1 Guernsey .......... .......... .......... .......... .......... .......... .......... .......... .......... ..........

B1 Le Conquet .......... .......... .......... .......... .......... .......... .......... .......... .......... ..........

B1 Ouessant .......... .......... .......... .......... .......... .......... .......... .......... .......... ..........

B1 Cherbourg .........G .......... .......... .......... .......... .......... .......... .......... .......... ..........

710 720 730 740 750 760 770 780 790 800

DWV-B TTAGCATGTC AGGTCTTATT ATGAATGCTC GAGTATTTAT TTCTGCGGTA GAGTAGGGAC CCCTCTATCT CTCAGGTACT GTATGAGGCG AAAGTGTGAA 796

DWV-A .......... ..A.TA.... ........GT T....G.A.. .......A.. ...CT..... .....AG... ........T. .......... .......... 795

B1 Penryn .......... .......... .......... .......... .......... .......... .......... .......... .......... ..........

B1 Liverpool .......... .......... .......... .......... .......... .......... .......... .......... .......... ..........

B1 Quiberon .......... .......... .......... .......... .......... .......... .......... .......... .......... ..........

B1 Arran .......... .......... .......... .......... .......... .......... .......... .......... .......... ..........

B1 Guernsey .......... .......... .......... .......... .......... .......... .......... .......... .......... ..........

B1 Le Conquet .......... .......... .......... .......... .......... .......... .......... .......... .......... ..........

B1 Ouessant .......... .......... .......... .......... .......... .......... .......... .......... .......... ..........

B1 Cherbourg .......... .......... .......... .......... .........G .......... .......... .......... .......... ..........

810 820 830 840 850 860 870 880 890 900

DWV-B AGTAATTTAT GTCTCTATAC ATAAGTGACT GTATCGGGAT TTCCTTTGGC AAGAATCCTT TTAATACAGT ATAATTTATG CCACGGTACG TTACGTTCGC 896

DWV-A ...TT.G... ..A.T.T..T ..GTAC.... .........A .......A.. .......... .......... .......G.. .T........ .......... 895

B1 Penryn .......... .......... .......... .......... .......A.. .......... .......... .....C.G.. .T........ ..........

B1 Liverpool .......... .......... .......... .......... .......... .......... .......... .......... .......... ..........

B1 Quiberon .......... .......... .......... .......... .......... .......... .......... .......... .......... ..........

B1 Arran .......... .......... .......... .......... .......... .......... .......... .......... .T........ ..........

B1 Guernsey .......... .......... .......... .......... .......... .......... .......... .......... .T........ ..........

B1 Le Conquet .......... .......... .......... .......... .......... .......... .......... .......... .......... ..........

B1 Ouessant .......... ....T..... .......... .......... .......... .......... .......... ......C... T......... ..........

B1 Cherbourg .......... .......... .......... .......... .......... .......... .......... .......... T......... ..........

910 920 930 940 950 960 970 980 990 1000

DWV-B AGGGCACCCG TTAATGTCAC ATAGCCCAGA CGATGACGAA TGGAAAGACA TTACTTTTTA TTTTAATGCT ACGATTATTG CTGTTTTATT TTGCTGTTTT 996

DWV-A .......... ........T. .......... .....G..G. .......... .C.TA..... .......... GTCT...... ...A...... .......... 995

B1 Penryn .......... ........T. .......... ........G. .......... .C.TA..... .......... GTCT...... ...A...... .....A....

B1 Liverpool .......... ........T. .......... .....G..G. .......... .C.TA..... .......... GTCT...... ...A...... ..........

B1 Quiberon .......... ........T. .......... .....G..G. .......... .C..A..... .......... GTCT...... ...A...... ..........

B1 Arran .......... .......... .......... ........G. .......... .C.TA..... .......... GTCT...... ...A...... ..........

B1 Guernsey .......... .......... .......... .......... .......... .......... .......... .......... .......... ..........

B1 Le Conquet .......... .......... .......... .......... .......... .......... .......... .......... .......... ..........

B1 Ouessant .......... .......... .......... .......... .......... .......... .......... .......... .......... ..........

B1 Cherbourg .......... .......... .......... .......... .......... .......... .......... .......... .......... ..........

1010 1020 1030 1040 1050 1060 1070 1080 1090 1100

DWV-B TATTTGCTAT TATAT-TTTG CTA-TTTCCA TTATTGCTAA ATATATTTCT TTGCTATTTT TGCTTTATAT ATTAGATTCA ATTCTTTTTA TTTTATATTT 1094

DWV-A .......... .T...A.... ...A...T.. .......G.. .......A.A .......... .AT.A....C GC........ ...T.A...T .CC....... 1095

B1 Penryn .......... .T...A.... ...A...T.. .......G.. .......A.A .......... .AT.A....C GC.C...... ...T.A...T .CC.......

B1 Liverpool C......... .T...A.... ...A...T.. .......G.. .......A.A .......... .AT.A....C GC........ ...T.A...T .CC.......

B1 Quiberon .......... .T...A.... ...A...T.. .......G.. .......A.A .......... .AT.A....C GC......TG ...T.A.... .CC.......

B1 Arran .......... .T...A.... ...A...T.. .......G.. .......A.A .......... .AT.A....C GC........ ...T.A...T .CC.......

B1 Guernsey .......... .....-.... ...-.A.T.. ....A..G.. ......A..A ....G..... ...A...... .CG....... C......A.. ........A.

B1 Le Conquet .......... .....-.... ...-...T.. .......... .......... .......... .......... .......... .......... ..........

B1 Ouessant .......... .....-.... ...-...T.. .......... .......... .......... .......... .......... .......... ..........

B1 Cherbourg .......... .....-.... ...-...T.. .......... .......... .......... .......... .......... .......... ..........

Breakpoint 1 (continued)

1110 1120 1130 1140 1150 1160 1170 1180 1190 1200

DWV-B TCAATTTGAT TTTGATTTTG AAGGTAAATA TATATAA--- --------A- AATGGCATTT AGTTGTGGAA CTCTTTCTTA TGCTGCTGTT GCCCAAGCTC 1182

DWV-A .......A.. .......... .......... .......TTA ATTATTAA.- ......C... .......... ....C..C.. CT....C..C .......... 1194

B1 Penryn .......A.. ........C. .......... .......TTA ATTATTGA.- ......C..C .......... .C........ CT....C... ..........

B1 Liverpool .......A.. ........C. .......... .......TTA ATCATTAAC- ......C... ..C....... .......... CT....C..C ..........

B1 Quiberon .......A.. ........C. .......... .......-TT AATTATTA.C ......C... ..C....... .......... CT....C..C ..........

B1 Arran .......A.. ........C. .......... .......TTA ATTATTAA.- ......C... .......... .......... CT....C..C ..........

B1 Guernsey C......A.. ......A.C. C...CG.... .......TWA ATTTTTAT.- ....TGT... ..A....... ....C...C

B1 Le Conquet .......... .......... .......... .......--- --------.- ......T... .AA....... ....CG.G.. CT.C...... ..W.TG....

B1 Ouessant C......... .......... .......... .......--- --------.- ......C... .......... .C..C..... CT....C..C ..........

B1 Cherbourg .......... .......... .......... .......--- --------.- ..AAAA..AA T.GMC.T..G W.G.GAAGCT CTA.C...C. CTG.CGT.G.

1210 1220 1230 1240 1250 1260 1270 1280 1290

DWV-B CCTCTGTAGC TCATGCTCCC CGTAGTTGGG AGATTGATGA AGCTAGGCGT CGACGCGTTA TTAAGCGTTT GGCGTTGGAA CAGGAACGGA 1272

DWV-A .G.....T.. CT....A..T ....CA.... .AGC...... .........G ..C..A..C. ....A..... ....C....G ..A.....T. 1284

B1 Penryn .A..C..C.. C.....A..T ....CA.... .AG....... .........G ..C..A..C. .C..A..... ....C....G ..A.....T.

B1 Liverpool .G.....C.. CT....A..T ....CG.... .AG....... .........G ..C..A.... ....A..... ....C....G ..A.....T.

B1 Quiberon .G.....T.. CTG...A..T ....CA.... .AG....... .........G ..C..A..C. ....A..... ....C....G ..A.....T.

B1 Arran .A.....C.. CT....A..T ....CA.... .AG.C..... .........G ..C..A..C. ....A..... ....C....G ..A.....T.

B1 Le Conquet .GC.C..T.T C.....R..K ....YR..CC .C..A..... GA.M...A.R ....TA..CG ..GCC.AGA. .T..C.C..G .G...CT.T.

B1 Ouessant .G.....C.. C.....A..T T...CA.... .AG....... .........G ..C..A..C. ....A..... ....C....G ..A.....T.

Breakpoint 2

1410 1420 1430 1440 1450 1460 1470 1480 1490 1500

DWV-B CTATAGCTGA AAGATGTACC CGCCGCCCTG TTCAAGAACA TGTCCCCATT TCAATCAGTA ATAGATATTC CCCTTTAGAA TCCCTTAAGA TTGAGGTAGG 1483

DWV-A .G..C..... .C.T.....G ..T..G...A .CA....G.. .TCT..T..A ...G.TTCG. ....G.T.G. T..AC.G... .........G .C.....C.. 1495

B2 Quiberon ---------- ---------- ---------- ---------- ---------- ---------- ---------- ---------- ---------G .C.....C.. 11

B2 Arran ---------- ---------- ---------- ---------- ---------- ---------- ---------- ---------- ---------G .C.....C.. 11

1510 1520 1530 1540 1550 1560 1570 1580 1590 1600

DWV-B AAAAGACGCG GGTGAGTTCG TGTTTAAGAA ACCCAAATAT ACAAAGATTT GTAAGAAAGT GAAACGGGTG GCATCAAAAT TTGTGCGCGA GAAAGTTGTT 1583

DWV-A TC....A..A ..C..A.GTA .A.C...... ...T...... ..GCGCG... .C........ ...G.AT..T ...A.TCGC. .C..T..T.. A......... 1595

B2 Quiberon TC....A..A ..C..A.GTA .A........ ...T...... ..GCGCG... .C........ ...G..T..T ...A.TCGC. .C..T..T.. A......... 111

B2 Arran TC....A..A ..C..A.GTA .A........ ...T...... ..GCGCG... .C........ ...G..T..T ...A.TCGC. .S.AT..T.. A......... 111

1610 1620 1630 1640 1650 1660 1670 1680 1690 1700

DWV-B AGGCCCGTTT GTAATCGATC GCCCATGTTA TTATTTAAAA TTAAGAAAGT AATATATGAT TTACATTTGT ATCGGTTACG GAAACAAGTT CGGCTTCTCA 1683

DWV-A C.T..TA.G. ..TC.A.... C..T...C.. ........GC ........A. T..T...... ..G..C..A. ..A.A...A. A.....GC.. A.AA.GT.G. 1695

B2 Quiberon C.T..TA.G. ..TC.A.... C..T...C.. ........GC ........A. T..T...... .....C..A. ..A.A..GA. A.....GC.. A.AA.GT.G.

B2 Arran C.T..TT.G. ..TC.A.... C..T...C.. .......... .......... .......... .......... .......... .......... ..........

1710 1720 1730 1740 1750 1760 1770 1780 1790 1800

DWV-B GACGCGAAAA ACAGCGTGAA TACGAGTTAG AGTGTGTTAC TAGTTTGCTA CAGCTATCTA ATCCTGTTTC AGCTAAACCT GAGATGGACA ATCCTAATCC 1783

DWV-A ....TC.... ......C..T ..T....... .......C.. ..A.C..T.. ..AT....G. ....G..GCA G..A.....A ........T. .C........ 1795

B2 Quiberon ....TC.... ......C..T ..T....... .......... .......... ..A....... .......... .......... ........T. ..........

B2 Arran .......... .......... .......... .......... .......... .......... ........C. .......... .......... ..........

1810 1820 1830 1840 1850 1860 1870 1880 1890 1900

DWV-B TGGTCCAGAT GGTGAAGGTG AAGTTGAATT AGCAAAGGAT AGTAATGTAG TATTAACTAC ACAACGTGAT CCTAGTACCT CTATTCCTGC TCCAACTAGT 1883

DWV-A A..A..T... ..C..G.... .......... ..A....... ........T. .T.....A.. T..G..A... ........A. .......A.. G..GGTG..C 1895

B2 Quiberon .......... .......... .......... ..A....... .......... .......... .......... .....C.... .......... ..........

B2 Arran .......... .......... .......... G.A....... .......... .......... .......... ..C..C..T. .......... ..........

1910 1920 1930 1940 1950 1960 1970 1980 1990 2000

DWV-B GTGAAGTGGA GTAGATGGAC TAGTAATGAT GTTGTGGATG ATTATGCCAC TATAACTTCG CGTTGGTATC AAATTGCCGA ATTTGTATGG TCAAAGGATG 1983

DWV-A ..A..A.... .......... .......... ..A..A.... ....C..... A..C..A..T ..A....... .G.....T.. ......T... .......... 1995

B2 Quiberon .......... .......... .......... .......... .......... ......C... .......... .G..C..... ......G... ..........

B2 Arran .......... .......... .......... .......... .......... .......... .......... .G........ ......C... ..........

2010 2020 2030 2040 2050 2060 2070 2080 2090 2100

DWV-B ATCCATTTGA TAAGGAATTG GCGCGTTTAA TTTTACCTCG AGCTTTGTTA TCTAGTATTG AGGCTAATTC TGACGCTATT TGTGATGTAC CTAATACTAT 2083

DWV-A .......... ......G..A ..A....... ....G..... T......... ........A. .......... ...T.....A ........G. .......... 2095

B2 Quiberon .......... .......... .......... .......... .......... .......... .......C.. .......... ........G. ..........

B2 Arran .......... .......... .....C.... .......... .......... .......... .......... .......... .......... ..........

2110 2120 2130 2140 2150 2160 2170 2180 2190 2200

DWV-B TCCGTTTAAG GTACATGCAT ATTGGCGTGG AGATATGGAA GTTCGAGTGC AGATTAACTC GAATAAATTC CAGGTTGGTC AATTACAGGC AACTTGGTAC 2183

DWV-A C..A...... .....C.... .......A.. C......... ...A....T. .A.....T.. A......... ..A....... ....G..A.. T........T 2195

B2 Quiberon .......... .......... .......... .......... .......... .......... .......... .......... .......... ..........

B2 Arran .......... .......... .......... .......... .......... .......... .......... .......... .......... .........T

2210 2220 2230 2240 2250 2260 2270 2280 2290 2300

DWV-B TATTCGGATC ATGAAAATTT GAATATCCAG ACGAAGCGAA GTGTGTATGG TTTTTCGCAT ATGGATCATG CTTTGATTAG CGCATCAGCG AGTAATGAAG 2283

DWV-A .......... .......... ......ATC. T.T...A... .C..T..... A.....A..A .......... .......... T..G.....A .......... 2295

B2 Quiberon .......... .......... .......... .......... .......... .......... .......... .......... .......... ..........

B2 Arran .......... .......... ......T... .......... .......... .......... .......... .......... .......... ..........

2310 2320 2330 2340 2350 2360 2370 2380 2390 2400

DWV-B CAAAATTAGT GATACCTTTT AAACACGTAT ATCCATTCTT ACCAACGCGT GTCGTTCCTG ATTGGACAAC TGGTATTCTT GATATGGGTA CCTTAAATAT 2383

DWV-A .......... T..T..A... ..G..T..T. .......T.. ......AA.A A.T..G..A. .......T.. ...C...T.A .........G .T..G..C.. 2395

B2 Quiberon .......... .......... .......... .......... .......... .......... .......... A......... .......... ..........

B2 Arran .......... .......... .....T.... .......... .......... .......... .......... .......... .......... ..........

2410 2420 2430 2440 2450 2460 2470 2480 2490 2500

DWV-B TCGTGTAATT GCTCCACTAC GTATGAGTGC GACGGGACCA ACCACTTGTA ATGTTGTAGT ATTTATTAAG TTAAATAATA GTGAATTCAC TGGTACTTCT 2483

DWV-A ...C...... .....CT... .G........ T..T..T... ..T..C.... ....C..C.. G........A ........C. .C..G..T.. A..G...... 2495

B2 Quiberon .......... ......T... .......... .......... .......... .......... .......... .......... .......... ..........

B2 Arran .......... .....GT... .......... .......... .......... .......... .......... .......... ...G...... ...A......

2510 2520 2530 2540

DWV-B TCTGGTAAGT TTTACGCGAA TCAAATTAGG GCAAAACCTG AAA 2526

DWV-A .......... ....T....G C.....C... ..G....... .G. 2538

B2 Quiberon .......... .......... .......... .......... ...

B2 Arran .......... .......... ......C... .......... ...

Breakpoint 3

5010 5020 5030 5040 5050 5060 5070 5080 5090 5100

DWV-B CCGCTATTCC AGAAGCACCC AATGCTGAAG CAGAGGAGGC CAGTGCCTGG GTATCCATTA TTTATAATGG TGTGTGTAAT ATGTTGAATG TAGCCGCTCA 5083

DWV-A .T..CG.... ......T... .......... .G........ A.....T... .......... .......... .......... ...C.T.... .G..T..... 5095

B3 Cherbourg .......... .......... .......... .......... .......... .......... .......... .......... .......... ..........

B3 Liverpool .......... .......... .......... .......... .......... .......... .......... .......... .......... ..........

B3 Guernsey .......... .......... .......... .......... .......... .......... .......... .......... .......... ..........

5110 5120 5130 5140 5150 5160 5170 5180 5190 5200

DWV-B AAAACCGAAA CAATTTAAAG ATTGGGTAAA ATTAGCTACC GTAGATTTTA GTAATAATTG TAGAGGTAGT AATCAGGTAT TTGTGTTTTT CAAGAATACG 5183

DWV-A .......... .......... .......... .........T .......... .......... .......... ..C....... ....A..... .........A 5195

B3 Cherbourg .......... .......... .......... .......... .......... .......... .......... ..C....... .......... ..........

B3 Liverpool .......... .......... .......... .......... .......... .......... .......... .......... .......... ..........

B3 Guernsey .......... .......... .......... .......... .......... .......... .......... .......... .......... ..........

5210 5220 5230 5240 5250 5260 5270 5280 5290 5300

DWV-B TTTGAAGTGT TGAAGAAAAT GTGGGGTTAT GTGTTTTGTC AGAGTAATCC TGCAGCGCGA CTCTTGAAAG CAGTGAATGA TGAACCTGAG ATTTTAAAAG 5283

DWV-A .......... .......... .......... ..A....... .......... .........C T.G....... .T........ C..G...... .....G.... 5295

B3 Cherbourg .......... .......... .......... .......... .......... .......... .......... .......... ...G...... .....G....

B3 Liverpool .......... .......... .......... .......... .......... .......... .......... .......... .......... ..........

B3 Guernsey .......... .......... .......... .......... .......... .......... .......... .......... .......... ..........

5310 5320 5330 5340 5350 5360 5370 5380 5390 5400

DWV-B CGTGGGTTAA AGAATGTCTG TATTTAGATG ATCCTAAATT TAGAATGCGA CGTGCGCATG ATCAAGAGTA TATTGAGAGA GTGTTTGCGG CCCATTCGTA 5383

DWV-A .A.....G.. G......... .....G.... .......... C........T ..A....... .......... .......... .......... .A.....A.. 5395

B3 Cherbourg .A.....G.. G......... .....G.... .......... .........T ..A....... .......... ...C...... .......... .A.....A..

B3 Guernsey .......... .......... .......... .......... C......... .......... .......... .......... .......... ..........

B3 Liverpool .......... .......... .......... .......... C......... .......... .......... ...C...... .......... ..........

5410 5420 5430 5440 5450 5460 5470 5480 5490 5500

DWV-B TGGACAAATT TTATTGCATG ACTTAACGGC TGAAATGAAT CAATCGCGTA ATTTAAGTGT GTTTACGAGA GTGTATGATC AAATATCTAA ATTGAAGACG 5483

DWV-A .......... ..GC.A.... .T.....T.. .......... .....A..A. ....G..... ......AC.C .......... ....T..A.. .........C 5495

B3 Cherbourg .......... ..GC.A.... .T.....T.. .......... .....A..A. .......... ......AC.T .......... ....T..A.. .........C

B3 Liverpool .......... .......... .......... .......... ........A. .......... .......... .......... .......... ......A..C

B3 Guernsey .......... .......... .......... .......... .......... .......... .......... .......... .......... ..........

5510 5520 5530 5540 5550 5560 5570 5580 5590 5600

DWV-B GATCTCATGG AAATGGGATC AAACCCATAT ATCAGGCGTG AATGCTTTAC GATTTGTATG TGTGGTGCAT CTGGAATTGG TAAGTCTTAT TTAACTGATT 5583

DWV-A .....T.... .......... G..T...... ..A....... ....T..... ...A..C... .......... .......... A..A..A... ..G..C.... 5595

B3 Cherbourg .....T.... .......... G..T...... ..A....... ....T..... ...A..C... .....G

B3 Liverpool .......... .......... ...T...... .......... .......... ...A..C... .....G

B3 Guernsey .......... .......... .......... .......... .......... ...A..C... .....G

References

Charon, J., Buchmann, J. P., Sadiq, S., & Holmes, E. C. (2022). RdRp-scan: A bioinformatic resource to identify and annotate divergent RNA viruses in metagenomic sequence data. *Virus Evolution, 8*(2). doi:10.1093/ve/veac082

Dobelmann, J., Manley, R., & Wilfert, L. (2024). Caught in the act: the invasion of a viral vector changes viral prevalence and titre in native honeybees and bumblebees. *Biology Letters, 20*(5), 20230600. doi:10.1098/rsbl.2023.0600

Dobelmann, J., & Wilfert, L. (2024). How population structure and sociality shape disease patterns in bumble bees. Authorea. November 07, 2024. *Authorea*. doi:10.22541/au.173098488.85952914

Edgar, R. C. (2004). MUSCLE: multiple sequence alignment with high accuracy and high throughput. *Nucleic Acids Research, 32*(5), 1792-1797.

Kearse, M., Moir, R., Wilson, A., Stones-Havas, S., Cheung, M., Sturrock, S., . . . Drummond, A. (2012). Geneious Basic: An integrated and extendable desktop software platform for the organization and analysis of sequence data. *Bioinformatics, 28*(12), 1647-1649. doi:10.1093/bioinformatics/bts199

Manley, R., Temperton, B., Doyle, T., Gates, D., Hedges, S., Boots, M., & Wilfert, L. (2019). Knock-on community impacts of a novel vector: spillover of emerging DWV-B from Varroa-infested honeybees to wild bumblebees. *Ecology Letters, 22*(8), 1306-1315. doi:10.1111/ele.13323
